# *Mycolicibacterium lacusdiani* sp. nov., an Attached Bacterium of *Microcystis aeruginosa*

**DOI:** 10.3389/fmicb.2022.861291

**Published:** 2022-05-12

**Authors:** Yao Xiao, Jian Chen, Min Chen, Shao-Ji Deng, Zhi-Qian Xiong, Bao-Yu Tian, Bing-Huo Zhang

**Affiliations:** ^1^College of Pharmacy and Life Science, Jiujiang University, Jiujiang, China; ^2^College of Life Science, Fujian Normal University, Fuzhou, China

**Keywords:** *Mycolicibacterium lacusdiani* sp. nov., attached bacteria, *Microcystis aeruginosa*, microcystin LR, coculture

## Abstract

In eutrophic water, attached bacteria of *Microcystis* play an important role in the formation, development, and degradation of *Microcystis* blooms. A novel actinobacterium, designated as JXJ CY 35^T^, was isolated from the culture mass of *Microcystis aeruginosa* FACHB-905 (Maf) collected from Lake Dianchi, Yunnan Province, China. Strain JXJ CY 35^T^ was gram-positive, acid-fast staining, aerobic, with short rod-shaped cells, positive for catalase, and negative for oxidase. The isolate was able to grow at 10.0–36.0°C, pH 4.0–10.0, and tolerate up to 5.0% (w/v) NaCl, with optimal growth at 28°C, pH 7.0–8.0, and 0% (w/v) NaCl. Cell-wall peptidoglycan contains aspartic acid, glutamic acid, glycine, and alanine, with mannose, ribose, galactose, and arabinose as whole-cell sugars. Polar lipids consist of diphosphatidylglycerol (DPG), phosphatidylethanolamine (PE), glycolipid (GL1-3), phosphoglycolipid (PGL), phosphatidylinositol (PI), and unidentified lipid (L1). The predominant menaquinone was MK-9. Major fatty acids (>10%) were C_17:1_ω7c (37.0%) and C_18:1_ω9c (18.9%). The complete genome sequence of strain JXJ CY 35^T^ was 6,138,096 bp in size with a DNA G + C content of 68.3%. Based on 16S rRNA gene sequences, it has 98.2% similarity to *Mycolicibacterium arabiense* JCM 18538^T^. The digital DNA-DNA hybridization (dDDH) and average nucleotide identity (ANI) values between strain JXJ CY 35^T^ and the closest five type strains *M. arabiense* JCM 18538^T^, *M. goodii* ATCC 700504^T^, *M. mageritense* DSM 44476^T^, *M. austroafricanum* DSM 44191^T^, and *Mycobacterium neglectum* CECT 8778^T^ were 52.1, 20.3, 20.3, 20.6, and 19.8%, and 92.7, 75.5, 75.6, 76.0, and 75.2%, respectively. On the basis of the above taxonomic data and differences in physiological characteristics from the closely related type strain, strain JXJ CY 35^T^ was determined to represent a novel species of genus *Mycolicibacterium*, for which the name *Mycolicibacterium lacusdiani* sp. nov., is proposed. The type strain is JXJ CY 35^T^ (=KCTC 49379^T^ = CGMCC 1.17501^T^). Different inoculation dosages of the type strain JXJ CY 35^T^ could exhibit different effects on the growth of Maf and its toxin synthesis and release. Strain JXJ CY 35^T^ could promote the growth of Maf by providing it with available phosphorus, nitrogen, probably vitamins, and plant growth hormones.

## Introduction

The attached bacteria of *Microcystis* play important roles in the occurrence, outbreak, and decline of *Microcystis* water bloom (Yang and Xiao, 2011). Many attached bacteria of *Microcystis* were isolated and identified, such as *Pseudomonas* (Zhou et al., 1998), *Sphingomonas* (Shao et al., 2014), *Erythrobacter* (Zhao et al., 2011), *Gordonia* and *Burkholderia* (Zhao et al., 2012), and many other *Proteobacteria, Bacteroidetes, Actinobacteria*, and *Deinococcus-Thermus* (Berg et al., 2009; Shao et al., 2014; Guo et al., 2020). *Microcystis aeruginosa* (Ma) is the most common species of bloom-causing cyanobacteria (Park et al., 2009) and the main producer of microcystins (MCs), a series of structurally similar hepatotoxins (Dawson, 1998). Ma belongs to the genus *Microcystis* and distributes worldwide.

Interactions between algae and attached bacteria are extremely complex and are categorized into the nutrient exchange, signal transduction, and gene transfer, with nutrient exchange being considered as the most common type of interaction (Kouzuma and Watanabe, 2015) and frequently the basis of algal–bacterial mutualism (Cooper and Smith, 2015). Algae provide attached bacteria with dissolved organic matters such as polysaccharides, and attached bacteria provide algae with various nutrients such as available phosphorus (P), nitrogen (N), and others (Yang and Xiao, 2011). However, the mutualism between algae and attached bacteria is mediated by the provision of nutrients from bacteria (Cooper and Smith, 2015).

Lake Dian (99°39′E, 22°23′N) is the sixth-largest and one of the most severely polluted freshwater lakes in China. During 1990–2005, cyanobacterial blooms occurred almost every year in this lake (Liu et al., 2006). Ma is the dominant bloom-forming species in Lake Dianchi (Liu, 1999). In our previous studies, many attached bacteria, including three novel species (Zhang et al., 2016a,b, 2017), were isolated from the culture mass of Ma isolated from Lake Dianchi (Guo et al., 2020). In this study, another novel actinobacterium strain JXJ CY 35^T^, belonging to the genus *Mycolicibacterium*, was isolated from the culture mass of *M. aeruginosa* FACHB 905 (Maf), which was isolated from the *Microcystis* water bloom sample collected from Lake Dianchi. The genus *Mycolicibacterium*, mainly from environmental samples, was divided from the genus *Mycobacterium* by Gupta et al. (2018) on the basis of phylogenomic and comparative genomic studies. At the time of writing, the genus *Mycolicibacterium* contains 90 validly named species. In order to understand the mutualistic symbiosis of strain JXJ CY 35^T^ and Maf, we confirmed its taxonomic status based on the multiphase analysis and studied their interaction by the coculture method.

## Materials and Methods

### Isolation and Maintenance of Strain JXJ CY 35^T^

*Microcystis aeruginosa* FACHB 905 culture was obtained from the Freshwater Algae Culture Collection at the Institute of Hydrobiology (FACHB collection), Chinese Academy of Sciences (Wuhan, China; http://algae.ihb.ac.cn/Default.aspx), and incubated in Hughes Gorham Zehnder (HGZ) medium as described previously (Zhang et al., 2015). To obtain pure cultures of attached bacteria, 0.2-ml aliquots of Maf culture were spread onto International Streptomyces Project 2 (ISP 2) medium and incubated at 28.0°C for 2–5 days. The isolated strain JXJ CY 35^T^ was stored on ISP 2 at 4.0°C and as glycerol suspensions (30%, v/v) at −80.0°C.

### Phenotypic Characteristics

Strain JXJ CY 35^T^ was cultured using ISP 2, tryptic soy agar (TSA), Middlebrook 7H10 agar, and Löwenstein–Jensen medium (Jensen, 1932) at 28.0°C. Cell morphology was observed using light microscopy (BX53; Olympus) and transmission electron microscope (JEM-2100, JEOL) from a culture grown on ISP 2 at 28.0°C for 3 days. Gram staining was done by using the standard Gram's staining procedure. Catalase activity was determined as described previously by Zhang et al. (2016b). Oxidase activity and motility were performed using standard methods (Kovacs, 1956; Xu et al., 2005). Acid-alcohol-fastness was examined according to the study by Dong and Cai (2001). Physiological and biochemical tests, including hydrolysis of Tweens (20, 40, and 80), starch and cellulose, and nitrate reduction, were done according to the study by Smibert and Krieg (1994). Anaerobic growth was determined after incubation on ISP 2 for 14 days at 28.0°C using the GasPak EZ Anerobe Pouch System (BD). Growth at different temperatures (5.0, 10.0, 15.0, 20.0, 28.0, 30, 32, 34.0, 36.0, 38.0, and 45.0°C), pH range (2.0–12.0 with interval of 1.0), and NaCl contents (0–10.0%, w/v, with interval of 1%) were tested using ISP 2 medium as the basal growth medium (Zhang et al., 2017). Other biochemical characteristics including various enzyme activities were further tested using the API ZYM, API 20NE, and API 50CH systems according to the manufacturer's instructions. Sensitivity to antibiotics was tested using a paper disk according to the method described by Zhang et al. (2016c).

### Chemotaxonomic Characterization

Cells of strain JXJ CY 35^T^ for chemical analysis were obtained from pure cultures grown on ISP2 agar at 28.0°C for 3 days. Polar lipids were extracted and analyzed according to the methods described by Minnikin et al. (1979). Fatty acid analysis was performed using the Sherlock Microbial Identification System (MIDI). Mycolic acids were analyzed using thin-layer chromatography (Minnikin et al., 1980). Menaquinones were extracted and analyzed according to the methods described by Tindall (1990). The peptide subunit of peptidoglycan and whole-cell sugars were detected according to the methods described by Hasegawa et al. (1983) and Tang et al. (2009).

### Phylogenetic and Genome Sequencing Analysis

Extraction of genomic DNA and PCR amplification of the 16S rRNA gene sequence were done as described by Peng et al. (2006) after being cultured on ISP 2 medium at 28.0°C for 3 days. The 16S rRNA gene sequence of the isolate was compared with sequences from EzBioCloud (https://www.ezbiocloud.net/) server databases (Yoon et al., 2017). Phylogenetic trees were generated by neighbor-joining (Saitou and Nei, 1987), maximum-parsimony (Fitch, 1971), and maximum-likelihood (Felsenstein, 1981) algorithms using MEGA version 5.0 (Tamura et al., 2011). The topologies of the resultant trees were evaluated using bootstrap analysis (Felsenstein, 1985) with 1,000 replications.

The genome sequencing, assembling, and annotation of strain JXJ CY 35^T^ were performed at Sangon Biotech (Shanghai, China) using the Illumina HiSeq 4000 platform. The genome was assembled using SPAdes version 3.5.0 (Bankevich et al., 2012) and corrected using PrInSeS-G. Gene prediction was performed using Prokka software tool (Seemann, 2014). Genomic annotation was performed using NCBI version Blast 2.2.28 with the default parameters. The digital DNA-DNA hybridization (dDDH) and average nucleotide identity (ANI) values between strain JXJ CY 35^T^ and the related strains were calculated using the Genome-to-Genome Distance Calculator (GGDC, version 3.0) (http://ggdc.dsmz.de/ggdc.php) (Meier-Kolthoff et al., 2013) and JSpeciesWS (JSWS) website (http://jspecies.ribohost.com/jspeciesws/#analyse), respectively. The genomes of type strains *M. arabiense* JCM 18538^T^, *M. goodii* ATCC 700504^T^, *M. mageritense* DSM 44476^T^, *M. austroafricanum* DSM 44191^T^, and *Mycobacterium neglectum* CECT 8778^T^ were downloaded from the NCBI genome database (https://www.ncbi.nlm.nih.gov/genome/).

### Abilities of Nitrogen-Fixing and Dissolving Unavailable Phosphate

The ability of nitrogen-fixing was determined by culturing in the nitrogen-free medium (glucose, 10 g; KH_2_PO_4_, 0.2 g; MgSO_4_·7H_2_O, 0.2 g; NaCl, 0.2 g; CaSO_4_·2H_2_O, 0.2 g; deionized water, 1,000 ml; pH, 7.0–7.2) at 28°C for 2 days, and the cell density was determined by spread-plate counting. The ability to dissolve insoluble phosphorus was determined according to the method described by Zhang et al. (2016b). Both Ca_3_(PO_4_)_2_ and phytin were used as the insoluble phosphorus at the dosages of 5 g/L.

### Coculture of Maf and Strain JXJ CY 35^T^

To understand the interactions between strain JXJ CY 35^T^ and Maf, we performed the following tests. A pure culture of Maf was obtained, examined, and cultured according to the methods described previously (Zhang et al., 2016b). Maf (about 2 × 10^6^ CFU/ml) was inoculated with strain JXJ CY 35^T^ at three different inoculums (1 × 10^5^, 1 × 10^6^, and 1 × 10^7^ CFU/ml). The pure culture of Maf served as the control. All of the coculture and controls were tested in triplicates and cultured, as described above, and sampled on days 5 and 10 of cultures. Bacterial densities of the samples were determined by the method of spread-plate counting. The contents of chlorophyll a (chl*-a*), extracellular microcystin LR (E-MC-LR), and intracellular microcystin LR (I-MC-LR) of the samples were determined according to the methods described previously (Zhang et al., 2015).

### Coculture of Maf and JXJ CY 35^T^ in the Absence of Available P and N

We performed the following tests to confirm if strain JXJ CY 35^T^ could promote the growth of Maf in the absence of available P and N. Maf (2 × 10^6^ CFU/ml) and strain JXJ CY 35^T^ (1 × 10^6^ CFU/ml) were cocultured using revised HGZ medium as described above. In the revised HGZ medium, KH_2_PO_4_ was replaced by Ca_3_(PO_4_)_2_, or NaNO_3_ was removed (nitrogen-free). The contents of chl-*a*, I-MC-LR, and E-MC-LR of the cultures, as well as the cell density of strain JXJ CY 35^T^ were determined as described above on day 12 of culture. Pure cultures of Maf (2 × 10^6^ CFU/ml) in the revised HGZ medium served as the controls.

## Results and Analysis

### Phenotypic Characteristics

**Figure 1:**
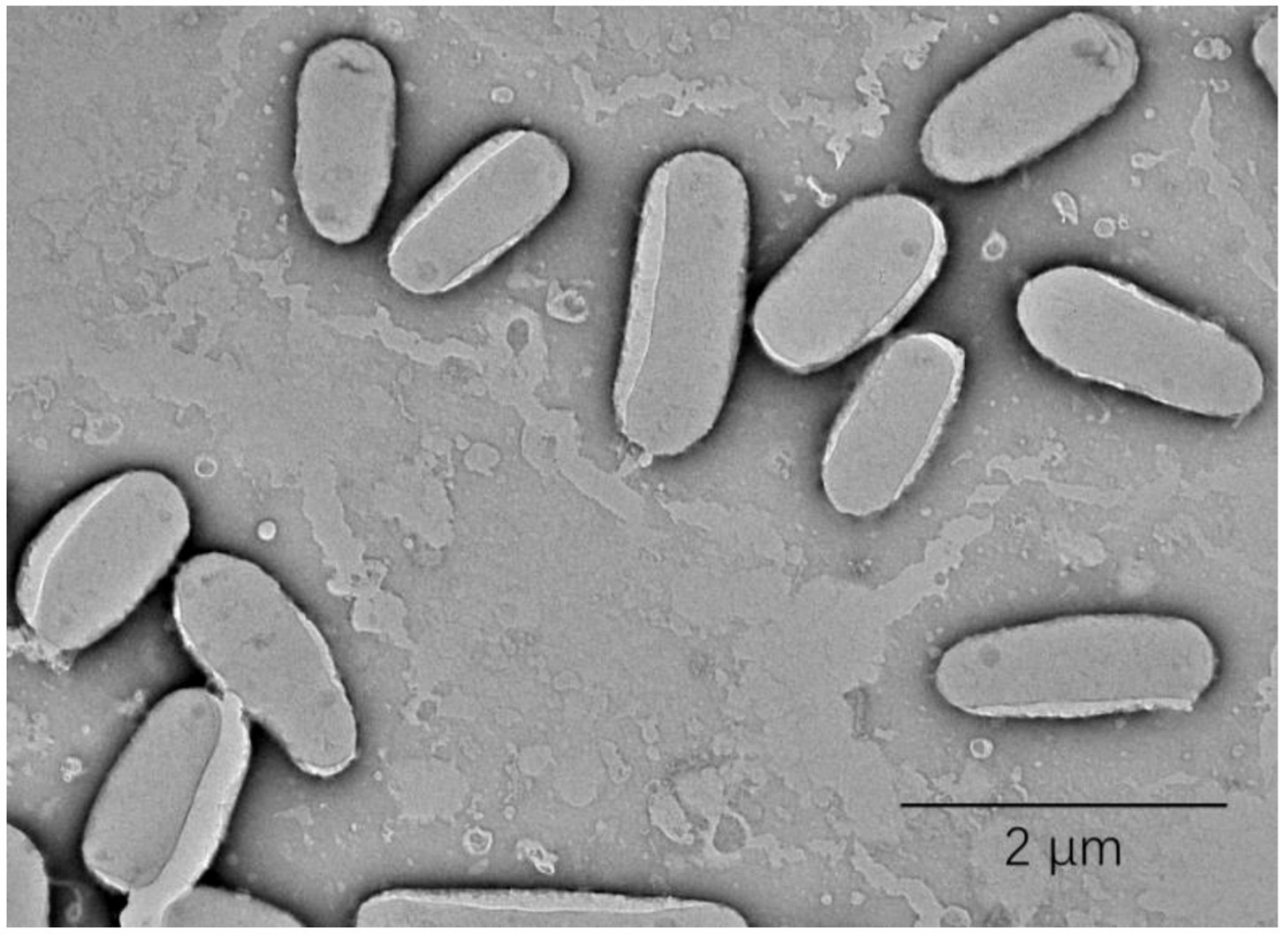
Transmission electron microscope image of strain JXJ CY 35^T^.

Strain JXJ CY 35^T^ was gram-positive, aerobic, and fast-growing. The colonies were orange in color, moist, smooth, and round. It grows well on ISP2 and TSA but poorly on Middlebrook 7H10 agar and Löwenstein–Jensen medium. The cells were short rods with a size of 0.6–0.9 × 1.2–2.4 μm (Figure 1). Growth occurred at 10.0–36.0°C, pH 4.0–10.0, and 0–5.0% (w/v) NaCl, with optimal growth at 28.0°C, pH 7.0–8.0, and 0% (w/v) NaCl. The isolate was found to be positive for catalase and negative for oxidase. It can hydrolyze Tweens (20, 40, and 80) and starch but cannot hydrolyze cellulose. Other differential phenotypic characteristics of strain JXJ CY 35^T^ and the related type strain *M. arabiense* DSM 45768^T^ are listed in Table 1, and the detailed characteristics are given in the type strain-description.

**Table 1:** Phenotypic and biochemical characteristics of strain JXJ CY 35^T^ and the reference strain.

**Characteristic**	**1**	**2**
Isolation source	Eutrophic fresh water	Coastal zone sand
Morphology	Rods	Rods
Colony color	Orange red	Orange red
Cell size (μm)	0.6–0.9 × 1.2–2.4	0.4–0.8 × 0.9–2.9
Temperature for growth (°C) (optimum)	10.0–36.0 (28.0)	5.0–37.0 (28.0–37.0)
pH range for growth (optimum)	4.0–10.0 (7.0–8.0)	6.0–8.0
Tolerance of NaCl (%, w/v) (optimum)	≤ 5 (0)	≤ 5 (0–2)
Nitrate reduction	+	–
Catalase	+	+
Oxidase	–	–
Hydrolysis of:		
Gelatin	–	–
Tween 20	+	+
Tween 40	+	+
Tween 80	+	+
Fatty acids (%)		
C_14:0_	5.4	4.0
C_16:0_	9.8	15.9
C_17:1_ω7c	37.0	17.5
C_18:1_ω9c	18.9	31.7
C_18:0_ 10-methyl	4.5	7.42
Summed feature 3*	14.9	15.9
DNA G+C content (%)	68.3	68.5

*1, JXJ CY 35^T^ (date from this study); 2, M. arabiense (Zhang et al., 2013). +, positive reaction; –, negative reaction; ND, no data. Summed features represent two or three fatty acids that cannot be separated by the MIDI. Summed feature 3 consisted of C_16:1_ ω7c/C_16:1_ ω6c*.

Strain JXJ CY 35^T^ was found to be sensitive to tetracycline (30.0 μg), rifampicin (5.0 μg), cefoxitin (30.0 μg), gentamicin (10.0 μg), kanamycin (30.0 μg), erythromycin (15.0 μg), neomycin (30.0 μg), chloramphenicol (30.0 μg), streptomycin (300.0 μg), and neomycin (5.0 μg), and it was resistant to ampicillin (10.0 μg), carboxylbenzicillin (100.0 μg), amoxicillin (10.0 μg), cefotaxidine (30.0 μg), and bacitracin (0.04 IU).

### Chemotaxonomic Characterization

The polar lipids of strain JXJ CY 35^T^ were found to be diphosphatidylglycerol (DPG), phosphatidylethanolamine (PE), glycolipid (GL1, 2, 3), phosphoglycolipid (PGL), phosphatidylinositol (PI), and unidentified lipid (L1) (Supplementary Figure S1). The mycolic acids showed obvious differences from those of the type strain *M. arabiense* DSM 45768^T^ (Supplementary Figure S2). Predominant cellular fatty acids (>10.0%) were identified as C_17:1_ω7c (37.0%) and C_18:1_ ω9c (18.9%). However, the main cellular fatty acids (>10.0%) of the reference strain *M. arabiense* DSM 45768^T^ were C_16:0_ (15.9%), C_17:1_ω7c (17.5%), and C_18:1_ω9c (31.7) (Table 1). The predominant menaquinone was found to be MK9. The peptidoglycan of strain JXJ CY 35^T^ contained aspartic acid, glutamic acid, glycine, and alanine, with mannose, ribose, galactose, and arabinose as whole-cell sugars.

### Phylogenetic Analysis

**Figure 2:**
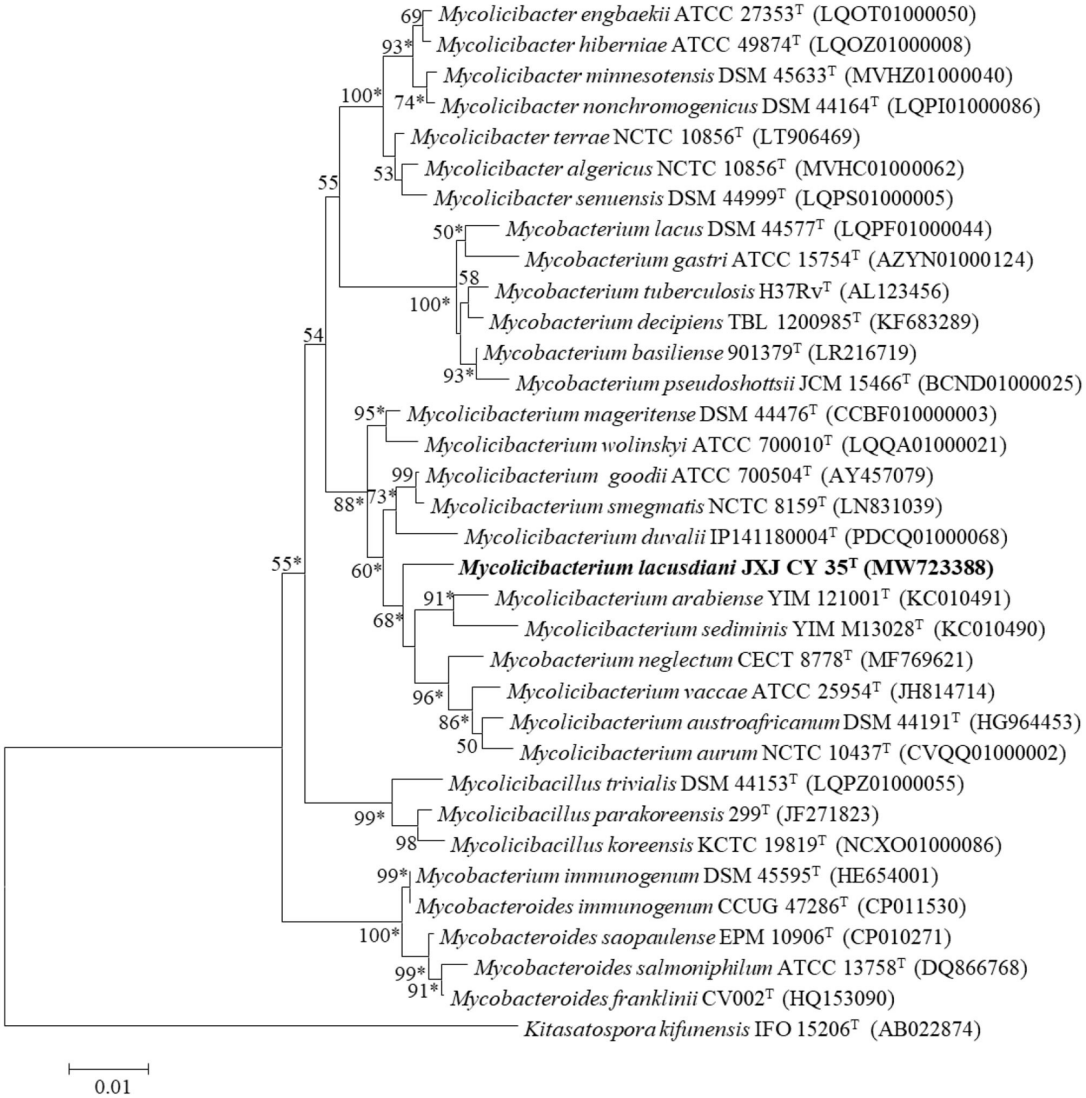
Neighbor-joining phylogenetic tree based on 16S rRNA gene sequences of strain JXJ CY 35^T^, its closest relative species of the genus *Mycolicibacterium*, and type strains of other genera. * Indicates clades that were conserved in neighbor-joining, maximum-likelihood, and maximum-parsimony trees. Bootstrap values (expressed as percentages of 1,000 replications) >50% are given at nodes. Scale bar: 0.01 sequence divergence.

Analysis of the almost-complete 16S rRNA gene sequence (1,514 bp; GenBank accession number: MW723388) indicated that strain JXJ CY 35^T^ is a member of the genus *Mycolicibacterium*. It shared 16S rRNA gene sequence similarities of 98.2% with *M. arabiense* DSM 45768^T^ (Zhang et al., 2013) and <98.2% with other members of the genus of *Mycolicibacterium*. The strain formed a distinct clade using three treeing methods (Figure 2; Supplementary Figures S3, S4). However, it formed a clade with *M. madagascariense* P2^T^ in three trees using all validly published species of the genus *Mycolicibacterium*. The digital DNA-DNA hybridization (dDDH) and ANI values between strain JXJ CY 35^T^ and the closest five type strains *Mycolicibacterium arabiense* JCM 18538^T^, *M. goodii* ATCC 700504^T^, *M. mageritense* DSM 44476^T^, *M. austroafricanum* DSM 44191^T^, and *Mycobacterium neglectum* CECT 8778^T^ were 52.1, 20.3, 20.3, 20.6, and 19.8%, and 92.7, 75.5, 75.6, 76.0, and 75.2%, respectively, based on the genomes from the NCBI genome database. Both values were lower than the species boundary recommended for distinguishing novel prokaryotic species (Kim et al., 2014; Chun et al., 2018). Therefore, based on the above data, it was concluded that strain JXJ CY 35^T^ represents a novel species of the genus *Mycolicibacterium*, for which the name *Mycolicibacterium lacusdiani* sp. nov. is proposed.

### Analysis of Genomic Characteristics

The genome of strain JXJ CY 35^T^ was sequenced and submitted to GenBank with the accession JAKCFC000000000. Its draft genome contains 24 contigs, with a total length of 6,138,096 bp and an N_50_ length of 596,067 bp. It has 5,946 protein-coding genes with a total size of 5,701,250 bp (an average of 958.8 bp per protein). Therefore, the coding ratio is 92.9%. The DNA G + C content was determined to be 68.3% based on the genome. No repeat region was found. The numbers of tRNA and rRNA are 50 and 3, respectively. According to the genome (6,017,160 bp; accession AP022593) obtained from the NCBI genome database, *M. arabiense* JCM 18538^T^ has some different genomic characteristics from strain JXJ CY 35^T^. It has 5,873 protein-coding genes with a total size of 5,598,840 bp (an average of 953.3 bp per protein). The coding ratio is 93.0%. The DNA G + C content is 68.5%. Unlike strain JXJ CY 35^T^, *M. arabiense* JCM 18538^T^ has 1,225 repeat regions with a total size of 116,611 bp and a repeat ratio of 1.94%. The numbers of tRNA and rRNA are 54 and 6, respectively. The gene annotation rates of strain JXJ CY 35^T^ and *M. arabiense* JCM 18538^T^ in various databases were diverse, which were about 98% in the TrEMBL and NR databases, but only about 41% in the Kyoto Encyclopedia of Genes and Genomes (KEGG) database (Supplementary Table S1). More genomic characteristics and gene annotation of strain JXJ CY 35^T^ are shown in Supplementary Figures S5–S9 and Supplementary Tables S1–S5.

Data from the genes annotated by the GO database showed that strain JXJ CY 35^T^ has three genes related to the arylsulfatase activity, indicating that this strain can potentially produce arylsulfatase. Data from COG database indicated that strain JXJ CY 35^T^ has a gene coding tellurite resistance protein and related permeases, indicating that it can grow in the presence of potassium tellurite. Strain JXJ CY 35^T^ has many genes or gene clusters in favor of coexisting with algae. Data from the genes annotated by the GO database showed that strain JXJ CY 35^T^ has 62 gene clusters related to symbiotic interaction, and immune response and their regulations (Supplementary Table S2), 34 genes related to phosphatases, 4 genes related to nodulation, 8 genes related to nitrogen fixation (Supplementary Table S3), 19 genes related to the synthesis of plant growth hormones (such as indole-3-acetic acid, auxin, abscisic acid, ethylene, and polyamine), 16 gene clusters related to the synthesis of various vitamins (Supplementary Table S4), 7 genes related to ATP-binding cassette (ABC) transporter complex, 8 genes related to protein secretion, and 29 gene clusters related to signaling and signaling pathway (Supplementary Table S5).

### Abilities of Nitrogen-Fixing and Dissolving Insoluble Phosphate

The cell density increased initially from 1.1 × 10^5^ to 3.1 × 10^6^ CFU/ml after 2 days of culture in a nitrogen-free medium, indicating that strain JXJ CY 35^T^ has the nitrogen-fixing ability. The contents of available phosphate were 1.6 ± 0.1 mg/L for Ca_3_(PO_4_)_2_ and 17.1 ± 0.3 mg/L for phytin on day 4 of culture, indicating that strain JXJ CY 35^T^ can dissolve insoluble phosphate.

### Influences of Coculture on the Growths of Maf and Strain JXJ CY 35^T^

**Figure 3:**
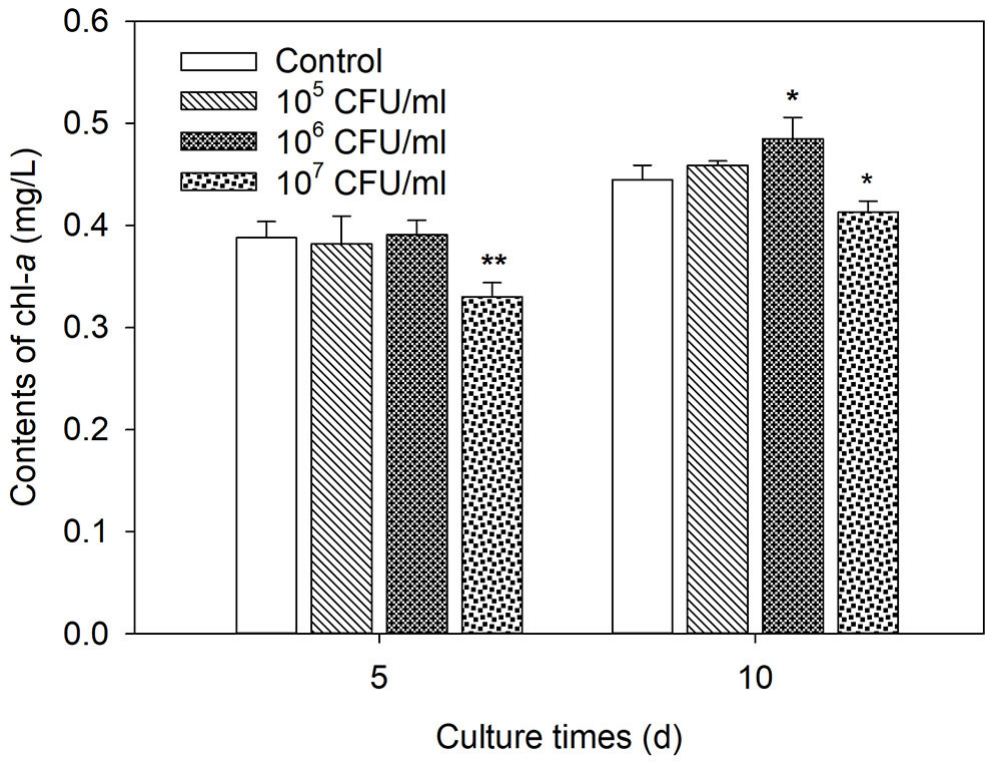
The influences of inoculation dosages and culture times of strain JXJ CY 35^T^ on the growth of Maf. The pure culture of Maf served as the control. Error bars indicate standard deviations for the three replicates. *, ** Indicate the significant differences between the control and the tests inoculated with different dosages of strain JXJ CY 35^T^ at the levels of *P* < 0.05 and *P* < 0.01.

The Chl-*a* contents of the control increased from initial 0.095 mg/L to 0.388 and 0.445 mg/L on days 5 and 10 of cultures, respectively. Strain JXJ CY 35^T^ showed no significant influence (*P* > 0.05) on the growth of Maf during the test at a low inoculation dosage (1 × 10^5^ CFU/ml). However, the chl-*a* contents of Maf increased by 9.0% (*P* < 0.05) on day 10 of culture at a moderate inoculation dosage (1 × 10^6^ CFU/ml) of strain JXJ CY 35^T^ and decreased by 14.9% and 7.2% (*P* < 0.05, *P* < 0.01) on days 5 and 10 of cultures, respectively, at a high inoculation dosage (1 × 10^7^ CFU/ml) of strain JXJ CY 35^T^ (Figure 3). Maf in cocultures probably exhibited influences on the growth of strain JXJ CY 35^T^. Cell densities of strain JXJ CY 35^T^ showed no significant difference (*P* > 0.05) between days 5 and 10 of cultures in a low inoculation dosage (1 × 10^5^ CFU/ml) group and decreased significantly (*P* < 0.01) on day 10 of culture in other two higher inoculation dosages (1 × 10^6^, 1 × 10^7^ CFU/ml) groups (Table 2).

**Table 2:** Changes in strain JXJ CY 35^T^ cell densities (CFU/ml) in coculture groups.

**Initial**	**On day 5**	**On day 10**
1 × 10^5^	1.64 ± 0.08 × 10^5^	2.02 ± 0.26 × 10^5^
1 × 10^6^	9.17 ± 0.87 × 10^5^	5.48 ± 0.47 × 10^5^^**^
1 × 10^7^	9.35 ± 0.57 × 10^6^	5.52 ± 0.25 × 10^6^^**^

** *Indicate the significant differences between days 5 and 10 of culture at the level of P < 0.01*.

### Influences of Coculture on Contents of MC-LR

**Figure 4:**
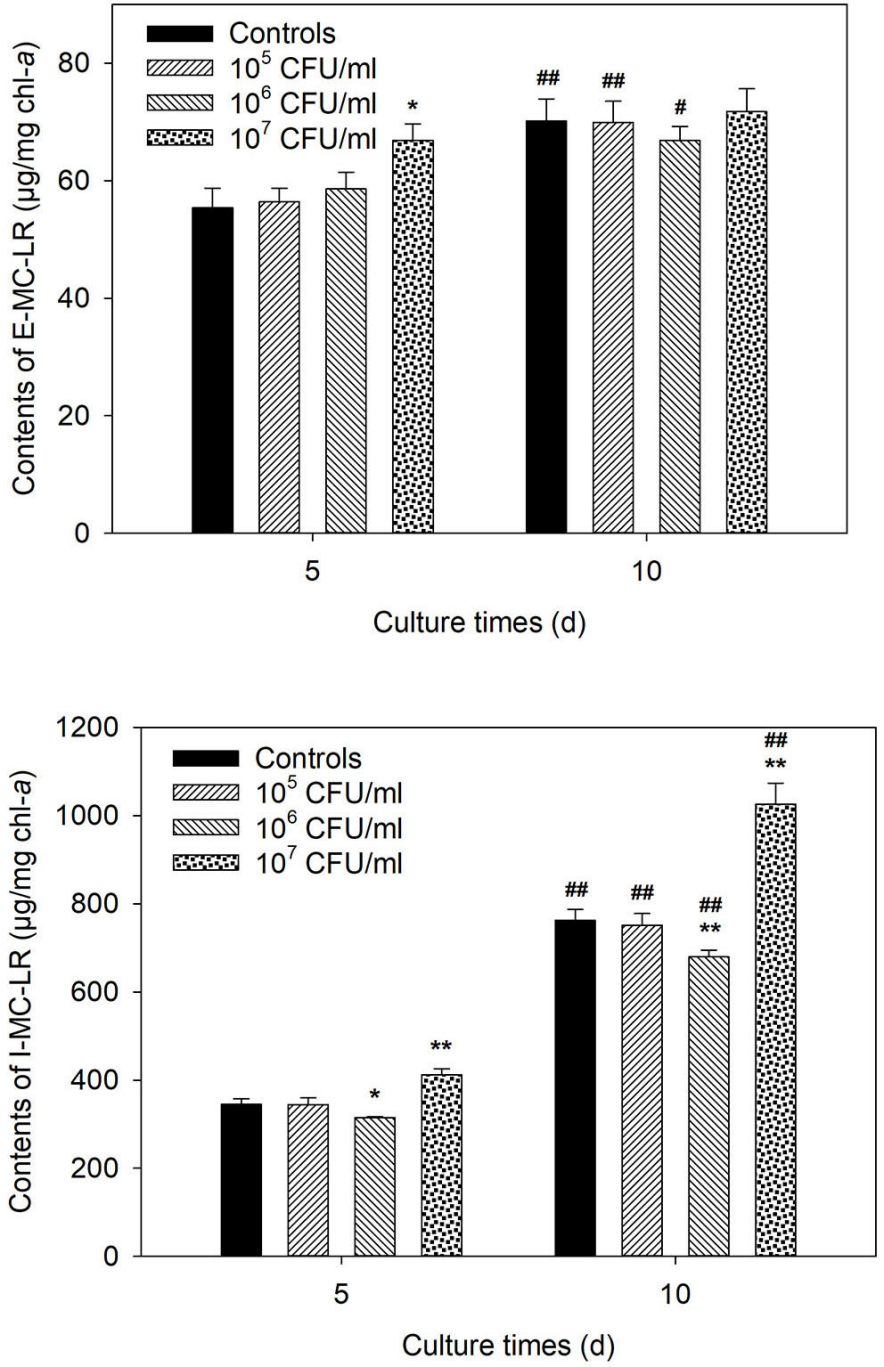
Influences of strain JXJ CY 35^T^ on the E-MC-LR and I-MC-LR of Maf at different inoculation dosages. *, ** Indicate the significant differences between the control and the tests inoculated with different dosages of strain JXJ CY 35^T^ at the levels of *P* < 0.05 and *P* < 0.01. #, ## Indicate the significant differences between days 5 and 10 of culture at the levels of *P* < 0.05 and *P* < 0.01.

Figure 4 shows that strain JXJ CY 35^T^ exhibited no significant influence on the E-MC-LR content of Maf at the inoculation dosages of both 1 × 10^5^ and 1 × 10^6^ CFU/ml during the test. However, the E-MC-LR content of the coculture group inoculated with 1 × 10^7^ CFU/ml of strain JXJ CY 35^T^ was 67.4 μg/mg chl-*a* on day 5 of culture (Figure 4), 21.6% higher than that of the control group (*P* < 0.01). Next, the E-MC-LR content of this coculture group did not increase significantly (*P* > 0.05) on day 10 of culture, unlike that of the control and the coculture groups inoculated with 1 × 10^5^ and 1 × 10^6^ CFU/ml of strain JXJ CY 35^T^ (*P* < 0.05, *P* < 0.01). Therefore, higher inoculation dosages of strain JXJ CY 35^T^ could promote the release of I-MC-LR temporarily.

Different inoculation dosages of strain JXJ CY 35^T^ exhibited different influences on the I-MC-LR content (Figure 4). Low inoculation dosage (1 × 10^5^ CFU/ml) showed no obvious influence on the I-MC-LR content of Maf in coculture. However, the I-MC-LR contents of moderate inoculation dosage (1 × 10^6^ CFU/ml) were 314.6 and 679.6 μg/mg chl-*a* on days 5 and 10 of cultures, which were 8.7% and 10.8% lower than that of the controls, respectively; the I-MC-LR contents of high inoculation dosage (1 × 10^7^ CFU/ml) were 411.9 and 1,026.1 μg/mg chl-*a* on days 5 and 10 of cultures, which were 19.5% and 34.6% higher than that of the controls, respectively.

### Influences of no Available N and P on Maf and JXJ CY 35^T^

In Ca_3_(PO_4_)_2_ medium, the chl-*a* content of Maf inoculated with JXJ CY 35^T^ was 0.170 mg/L (Table 3) on day 12 of culture, which were 13.3% higher than that of the control group (*P* < 0.05). In a nitrogen-free medium, the chl-*a* content of Maf inoculated with JXJ CY 35^T^ was 0.218 mg/L (Table 3), which were 27.5% higher than that of the control group (*P* < 0.01). Both E-MC-LR and I-MC-LR contents of Maf were significantly influenced (*P* < 0.01) by strain JXJ CY 35^T^ in the absence of available N and P (Table 3). In Ca_3_(PO_4_)_2_ medium, the contents of E-MC-LR and I-MC-LR were 235.0 and 625.0 μg/mg chl-*a* in the group inoculated with JXJ CY 35^T^, which were 15.6 and 43.3% lower (*P* < 0.01) than that of the control group, respectively. In a nitrogen-free medium, the contents of E-MC-LR and I-MC-LR were 109.2 and 439.5 μg/mg chl-*a* in the group inoculated with JXJ CY 35^T^, which were 32.4 and 29.9% lower (*P* < 0.01) than that of the control group, respectively.

**Table 3:** Influences of no available N and P on Maf and JXJ CY 35^T^ of cocultures.

**Groups**	**Contents of chl-*a* (mg/L)**	**Density of JXJ CY 35^**T**^ (CFU/ml)**	**Contents of MC-LR (μg/mg chl-** ***a*** **)**	
			**Intracellular**	**Extracellular**
Control-P	0.150 ± 0.001		1,101.9 ± 81.6	278.4 ± 3.7
Test-P	0.170 ± 0.010*	4.22 ± 027 × 10^5^	625.0 ± 11.8**	235.0 ± 5.5**
Control-N	0.171 ± 0.004		627.5 ± 10.2	161.6 ± 12.7
Test-N	0.218 ± 0.005**	5.87 ± 0.62 × 10^5^	439.5 ± 9.7**	109.2 ± 3.7**

Control-P, Ca_3_(PO_4_)_2_ medium inoculated with no strain JXJ CY 35^T^; Test-P, Ca_3_(PO_4_)_2_ medium inoculated with strain JXJ CY 35^T^; Control-N, nitrogen-free medium inoculated with no strain JXJ CY 35^T^; Test-N, nitrogen-free medium inoculated with strain JXJ CY 35^T^. ^*^, ^**^ Indicate the significant differences between the control and the tests inoculated with strain JXJ CY 35^T^ at the levels of *P* < 0.05 and *P* < 0.01.

### Description of *Mycolicibacterium Lacusdiani* sp. nov.

*Mycolicibacterium lacusdiani* sp. nov. (la.cus.di.a'ni L. gen. n. lacus, of a lake; N.L. gen. n. diani, of Dian; N.L. gen. n. lacusdiani, of Dian lake).

Strain JXJ CY 35^T^ is gram-positive, aerobic, fast-growing, nonmotile, and short rod-shaped (0.6–0.9 × 1.2–2.4 μm) and grows well on ISP 2 and TSA. The pH, NaCl content (w/v), and temperature range for growth are 4.0–10.0, 0–5.0%, and 10–36°C, respectively, with optimal growth at 7.0–8.0, 0% (w/v), and 28°C. It is positive for hydrolysis of Tweens (20, 40, and 80), nitrate reduction, and catalase and negative for hydrolysis of cellulose and oxidase. The diagnostic diamino acids in the cell-wall peptidoglycan were aspartic acid, glutamic acid, glycine, and alanine. MK9 was the predominant menaquinone, and polar lipids include diphosphatidylglycerol (DPG), phosphatidylethanolamine (PE), glycolipid (GL 1, 2, 3), PI, PGL, and L1. Major cellular fatty acids (>10.0%) were C_17:1_ω7c (37.0%) and C_18:1_ ω9c (18.9%).

The type strain, JXJ CY 35^T^ (=KCTC 49379^T^ = CGMCC 1.17501^T^), was isolated from the culture mass of *M. aeruginosa* FACHB-905 collected from Lake Dian Yunnan Province, south-west PR China, and has a DNA G + C content of 68.3%. The GenBank accession number for the 16S rRNA gene sequence and draft genome sequence of strain JXJ CY 35^T^ are MW723388 and JAKCFC000000000, respectively.

## Discussion

The genus *Mycobacterium* was divided into five genera, including an emended genus *Mycobacterium*, and four novel genera, namely, *Mycolicibacterium* gen. nov., *Mycolicibacter* gen. nov., *Mycolicibacillus* gen. nov., and *Mycobacteroides* gen. nov. by Gupta et al. (2018) on the basis of phylogenomic and comparative genomic studies of 150 out of 188 type strains. Moreover, there were other novel species belonging to the genus *Mycobacterium* being published (Nouioui et al., 2018) at the same time. Therefore, the taxonomic statuses of more than 38 species belonging to the emended genus *Mycobacterium* must be confirmed. This is probably the partial reason that some validly published species belonging to the genus *Mycobacterium* are close to strain JXJ CY 35^T^, such as *M. neglectum* CECT 8778^T^, *M. grossiae* SCH^T^, *M. aquaticum* RW6^T^, *M. lehmannii* SN 1900^T^, and *M. neumannii* SN 1904^T^, which did not appear in the article by Gupta et al. (2018). The dDDHs and ANIs between strain JXJ CY 35^T^ and the five type strains are 19.8–22.9% and 75.2–79.4%, based on the genomes from the NCBI genome database.

Algae and bacteria have coexisted ever since the early stages of evolution (Ramanan et al., 2016). Specific algae release distinct organic matters, which concludes that only specific bacteria can live and persist in the specific phycosphere (Yang et al., 2017). Many interactions, such as nutrient exchange, signal transduction, gene transfer, and inhibition, were formed between algae and bacteria during the long evolution (Kouzuma and Watanabe, 2015; Cirri and Pohnert, 2019). Analysis of metagenomes, metaproteomes, and metatranscriptomes undoubtedly contributed greatly to the recognition of the interactions between phytoplankton and heterotrophic bacteria (Kazamia et al., 2016). However, many aspects of these interactions (Grossart and Simon, 2007), as well as the physiological and biochemical characteristics and ecological functions of most bacteria (Zhang et al., 2019), are still unknown because most of the studies were performed under non-axenic conditions (Grossart and Simon, 2007). Cocultures of defined combinations of known and well-characterized partners in the laboratory can provide insight into the interactions at the molecular and cellular levels (Kazamia et al., 2016). We can know these potential interactions between algae and its specific attached bacterium by coculture method.

Available N and P in eutrophic water are the two key elements inducing cyanobacterial blooms. Our study indicated that strain JXJ CY 35^T^ can release available phosphate from both organic and inorganic insoluble P, which is consistent with its gene annotation from GO database (Supplementary Table S3). Therefore, similar to other attached bacteria (Yang and Xiao, 2011; Zhang et al., 2016a,b, 2017), strain JXJ CY 35^T^ could dissolve insoluble tricalcium phosphate, and further provided Maf with available phosphate for growth in the absence of available P (Table 3). Strain JXJ CY 35^T^ can grow in the nitrogen-free medium, indicating that it has N fixing activity and can convert N_2_ into NH_3_. This was proved by the data from the genes annotated by the GO database (Supplementary Table S3). Ammonia is another key factor in microbial interactions in the aquatic environment (Cirri and Pohnert, 2019). The genus *Microcystis* prefers ammonium (NH${}_{4}^{+}$-N) because of its attached bacteria lacking functional genes that mediate the nitrification process (Yang et al., 2021). Therefore, JXJ CY 35^T^ can promote the growth of Maf in the absence of available N.

The coculture results (Figure 3) indicated that opportune inoculation dosage of strain JXJ CY 35^T^ could promote the growth of Maf even if there was sufficient available P and N. Therefore, strain JXJ CY 35^T^ probably produces some other growth promoters. Similarly, strains of *Azospirillum* spp. (de-Bashan et al., 2008) and *Citricoccus lacusdiani* JXJ CY 21^T^ (Zhang et al., 2016b) could promote algae growth by indole-3-acetic acid and unknown substances, respectively, even when sufficient available P and N were provided. Attached bacteria can promote algae growth by providing them with vitamins (Cooper and Smith, 2015; Kouzuma and Watanabe, 2015). *Microcystis* requires vitamin B_12_ for the methionine biosynthesis pathway and other vitamins for growth (Hoke et al., 2021). The data of annotated genes (Supplementary Table S4) from the GO database indicated that strain JXJ CY 35^T^ could potentially secrete many plant growth hormones, such as indole-3-acetic acid, auxin, abscisic acid, ethylene, and polyamine, and various fat-soluble and water-soluble vitamins, such as vitamins B_1_, B_2_, B_6_, B_12_, K_1_, K_2_, and E, which may also be the growth-promoting factors of Maf under certain conditions. Organic nutrients such as organic carbon sources secreted by Ma (Casamatta and Wickstrom, 2000) can be utilized by attached bacteria for survival. β-glucosidase produced by bacteria is involved in utilizing organic carbon secreted by *Microcystis* (Yang et al., 2021). The data of annotated genes by the GO database indicated that strain JXJ CY 35^T^ has a gene cluster or three genes related to β-glucosidase (Supplementary Table S4). It also has glycoside hydrolases, carbohydrate esterases, and polysaccharide lyase (Supplementary Figure S9). Therefore, strain JXJ CY 35^T^ can use extracellular polysaccharides secreted by Maf as a carbon source.

The protein secretion system and ATP-binding cassette (ABC) transporter are involved in the exchange of substances and signal transduction among cyanobacteria and their attached bacteria (Zhu et al., 2021). Therefore, the exchange of substances and signal transduction, in fact, exist between Maf and strain JXJ CY 35^T^ based on the data of annotated genes (Supplementary Table S5) by the GO database. Moreover, two genes and gene clusters related to modulations of signal transduction and signal transduction pathways are involved in symbiotic interaction (Supplementary Table S5), which further supports the existence of signal transduction among Maf and strain JXJ CY 35^T^.

High inoculation dosages of strain JXJ CY 35^T^ could inhibit the growth of Maf (Figure 3), similar to *Pseudomonas* sp. X (Yang and Xiao, 2011), *Modestobacter lacusdianchii* JXJ CY 19^T^ (Zhang et al., 2016a), and *Citricoccus lacusdiani* JXJ CY 21^T^ (Zhang et al., 2016b). Attached bacteria of high cell densities probably compete with Maf for nutrients, which may further inhibit the growth of Maf over a period of time. However, the inhibition ratio decreased with the culture time and the decreasing cell density of the bacteria (Figure 3; Table 2), similar to other reports (Zhang et al., 2016a,b). The results (Tables 2, 3) showed that the cell density of strain JXJ CY 35^T^ could be maintained at about 10^5^ CFU/ml in cocultures with Maf regardless of the initial inoculation dosages of the bacteria. Therefore, healthy Maf may be able to control attached bacterial cell density to avoid competition for nutrients, similar to healthy macroalgae (Kouzuma and Watanabe, 2015). MCs show inhibition of many bacteria and probably play an important ecological role in eliminating competitors (Valdor and Aboal, 2007). However, we found that higher content of E-MC-LR (the unit was converted to μg/L from μg/mg chl-*a*) did not result in a lower cell density of strain JXJ CY 35^T^. Therefore, Maf may still have other unknown mechanisms to control the cell densities of attached bacteria.

The data (Figures 3, 4; Tables 2, 3) showed that the content of MC-LR decreased when the growth of Maf was promoted and increased when the growth of Maf was inhibited. Similarly, Schmidt et al. (2020) also reported that attached bacteria could promote the growth of Ma and reduce the synthesis of MCs. Since MCs may play an important ecological role in eliminating competitors, it is not difficult to understand that the synthesis of MCs probably is downregulated by conditions conducive to Maf growth and upregulated by conditions injurious to Maf growth. This probably partially explains that inoculation dosages of 1 × 10^6^ and 1 × 10^7^ CFU/ml strain JXJ CY 35^T^ resulted in the decrease and increase of I-MC-LR in Maf, respectively.

## Data Availability Statement

The data presented in the study are deposited in the GenBank repository, accession numbers MW723388 for the 16S rRNA gene sequence and JAKCFC000000000 for the draft genome sequence of strain JXJ CY 35T.

## Author Contributions

YX, JC, MC, S-JD, and Z-QX carried out the experiments. YX, JC, and B-HZ analyzed the data and prepared the manuscript. B-HZ designed the research. All authors contributed to the study and approved the submitted version.

## Funding

This study was supported by the National Natural Science Foundation of China (No. 31060010) and the Program of Jiujiang University (No. 201511).

## Conflict of Interest

The authors declare that the research was conducted in the absence of any commercial or financial relationships that could be construed as a potential conflict of interest.

## Publisher's Note

All claims expressed in this article are solely those of the authors and do not necessarily represent those of their affiliated organizations, or those of the publisher, the editors and the reviewers. Any product that may be evaluated in this article, or claim that may be made by its manufacturer, is not guaranteed or endorsed by the publisher.

## Abbreviations

Chl*-a*: chlorophyll*a*
DPG: diphosphatidylglycerol
E-MC-LR: extracellular microcystin LR
GL: glycolipid
I-MC-LR: intracellular microcystin LR
L1: unidentified lipid
Ma: *Microcystis aeruginosa*
Maf: *Microcystis aeruginosa* FACHB-905
PE: phosphatidylethanolamine
PGL: phosphoglycolipid
PI: phosphatidylinositol.
