# Supplementary material for: Mycolicibacterium lacusdiani sp. nov., an Attached Bacterium of Microcystis aeruginosa
Source: Front Microbiol. 2022 May 12;13:861291. doi: 10.3389/fmicb.2022.861291 (PMC9134240; doi:10.3389/fmicb.2022.861291)
Supplement: Supplementary file 1 [file Data_Sheet_1.pdf]

Mycolicibacterium lacusdiani sp. nov., an associated bacteria of *Microcystis aeruginosa*

Yao Xiao[1,2†], Jian Chen[1†], Min Chen[1], Shao-Ji Deng[1], Zhi-Qian Xiong[1], Bao-Yu Tian[2] and Bing-Huo Zhang[1*](✉)

[1]*College of Pharmacy and Life Science, Jiujiang University, Jiujiang, 332000, People's Republic of China*
[2]*College of Life Science, Fujian Normal University, Fuzhou, 350117, People's Republic of China*

Author for correspondence:
Bing-huo Zhang
Tel & Fax: +86-792-8565939
E-Mail: binghuozh@126.com
† These authors have contributed equally to this work and share first authorship

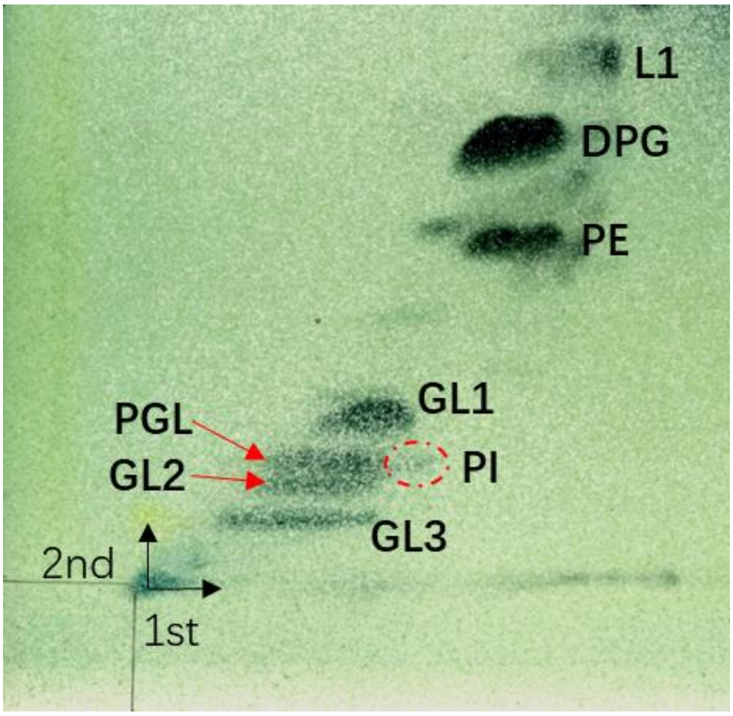

FIGURE S1 | The polar lipids of strain JXJ CY 35^T as revealed by two-dimensional TLC. DPG, diphosphatidylglycerol; GL1, glycolipid 1; GL2, glycolipid 2; GL3, glycolipid 3; L1, unidentified lipid; PE, phosphatidylethanolamine; PGL, phosphoglycolipid; PI, phosphatidylinositol.

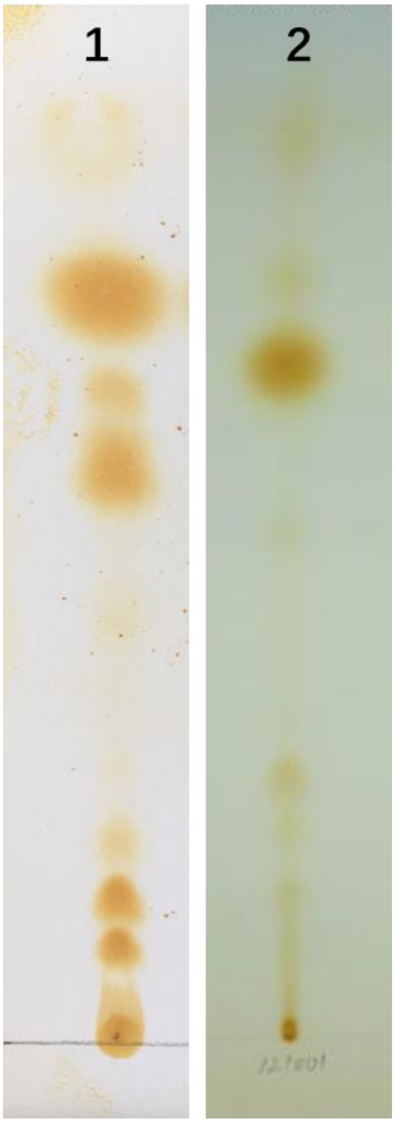

FIGURE S2 | One dimensional thin-layer chromatography of mycolic acids of strains JXJ CY 35^{T} (1) and *Mycolicibacterium arabiense* DSM 45768^{T} (2; Zhang, et al., 2013). Solvent: petroleum ether (b, p. 60-80°C)/acetone (95:5, v/v); staining agent: I_2.

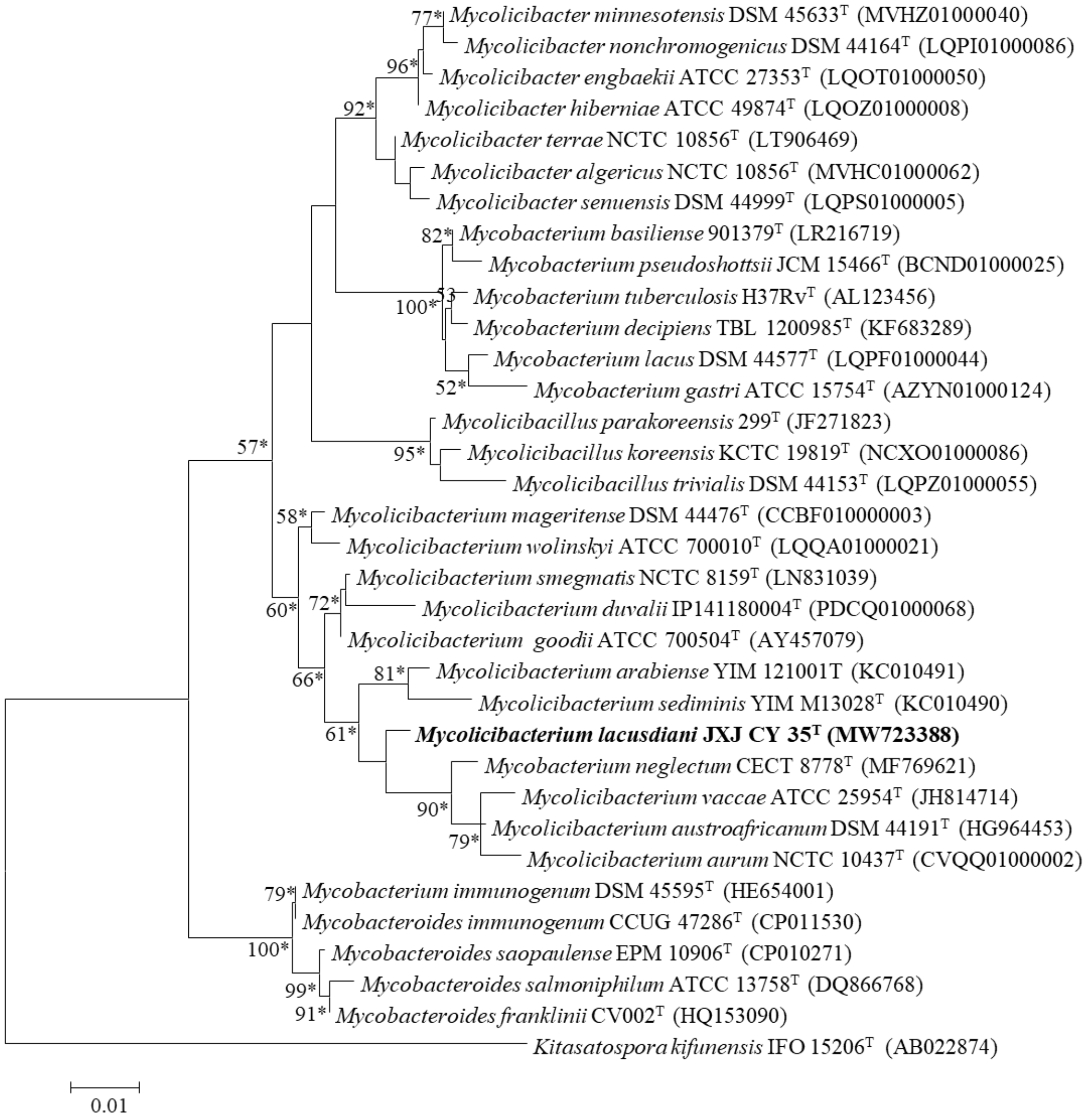

FIGURE S3 | Maximum-likelihood phylogenetic tree based on 16S rRNA gene sequences of strain JXJ CY 35^T and its closest relative species of the genus *Mycolicibacterium*. * indicate clades that were conserved in neighbour-joining, maximum-likelihood and maximum-parsimony trees. Bootstrap values (expressed as percentages of 1000 replications) > 50 % are given at nodes. Bar, 0.01 sequence divergence.

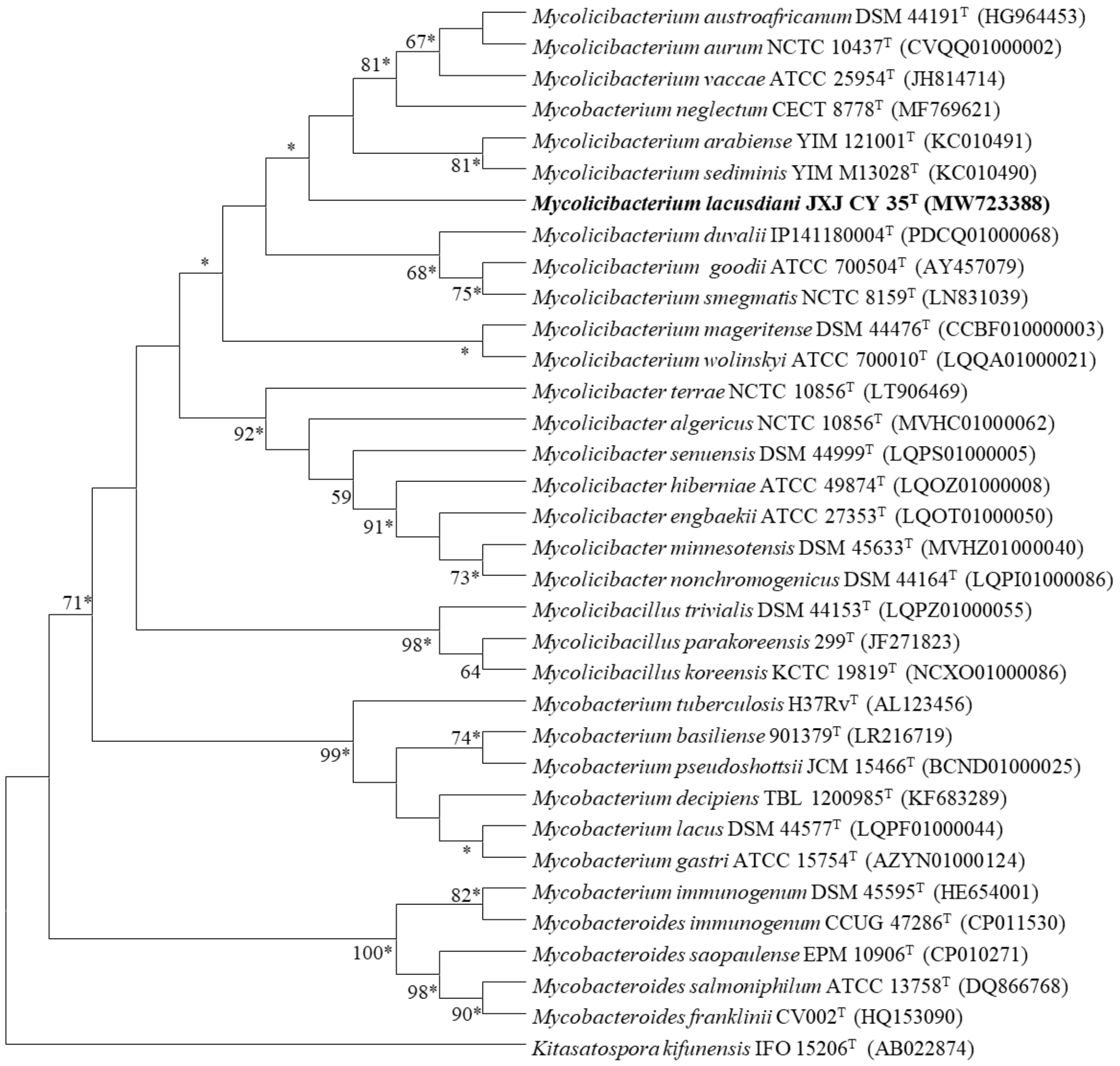

FIGURE S4 | Maximum-parsimony phylogenetic tree based on 16S rRNA gene sequences of strain JXJ CY 35^{T} and its closest relative species of the genus *Mycolicibacterium*. * indicate clades that were conserved in neighbour-joining, maximum-likelihood and maximum-parsimony trees. Bootstrap values (expressed as percentages of 1000 replications) > 50% are given at nodes.

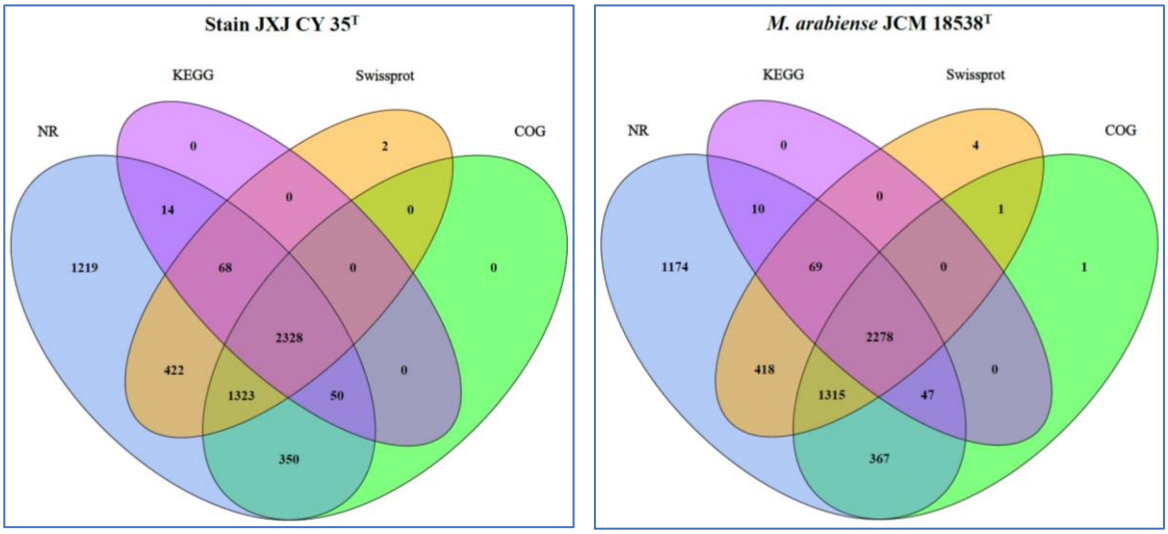

FIGURE S5 | Venn diagrams for gene annotations in different databases based on the genomes of stains JXJ CY 35^{T} and *M. arabiense* JCM 18538^{T}. (All data were from this study).

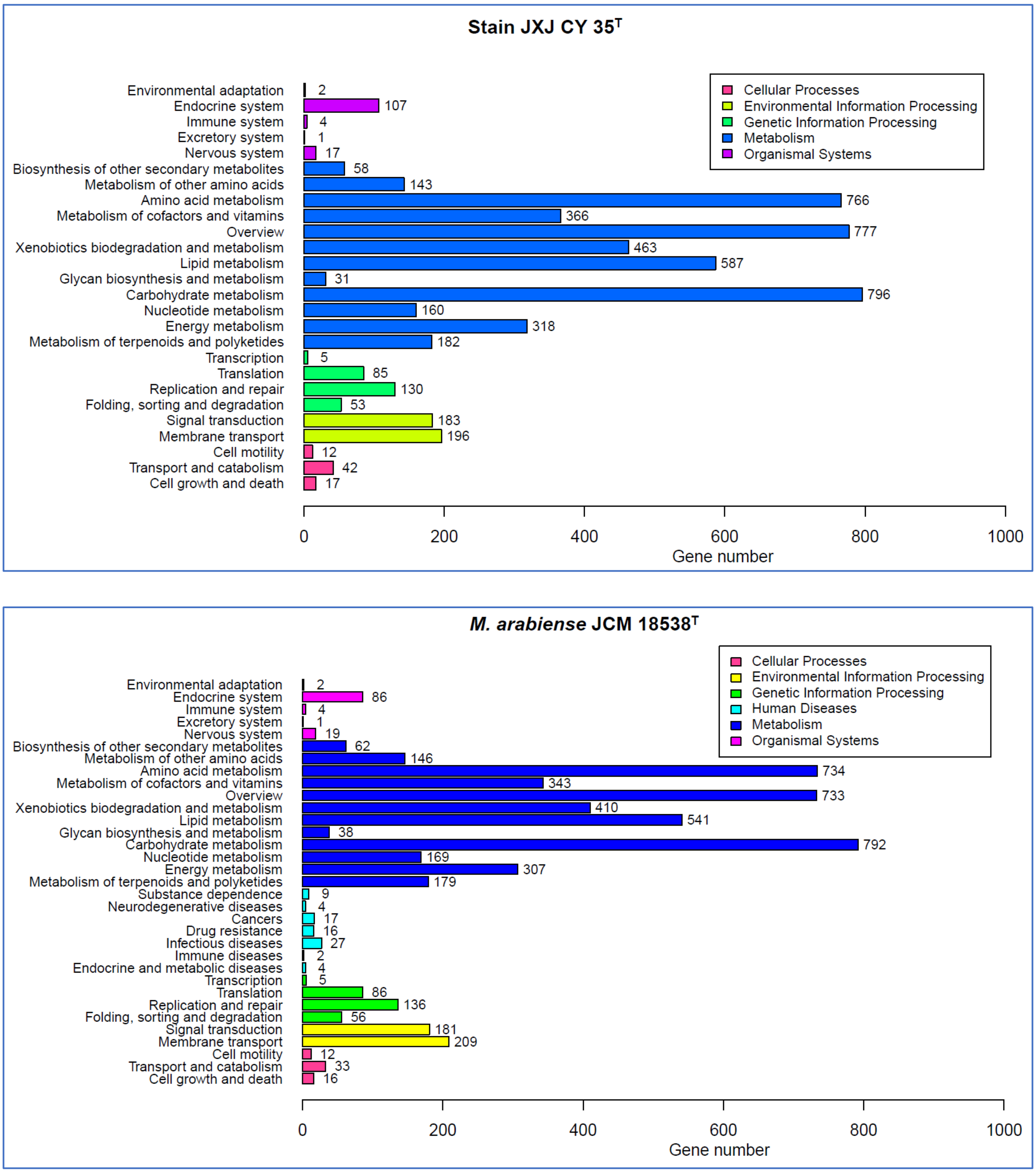

FIGURE S6 | Analysis of the KEGG functional classifications based on the genomes of stains JXJ CY 35^T and *M. arabiense* JCM 18538^T. (All data were from this study).

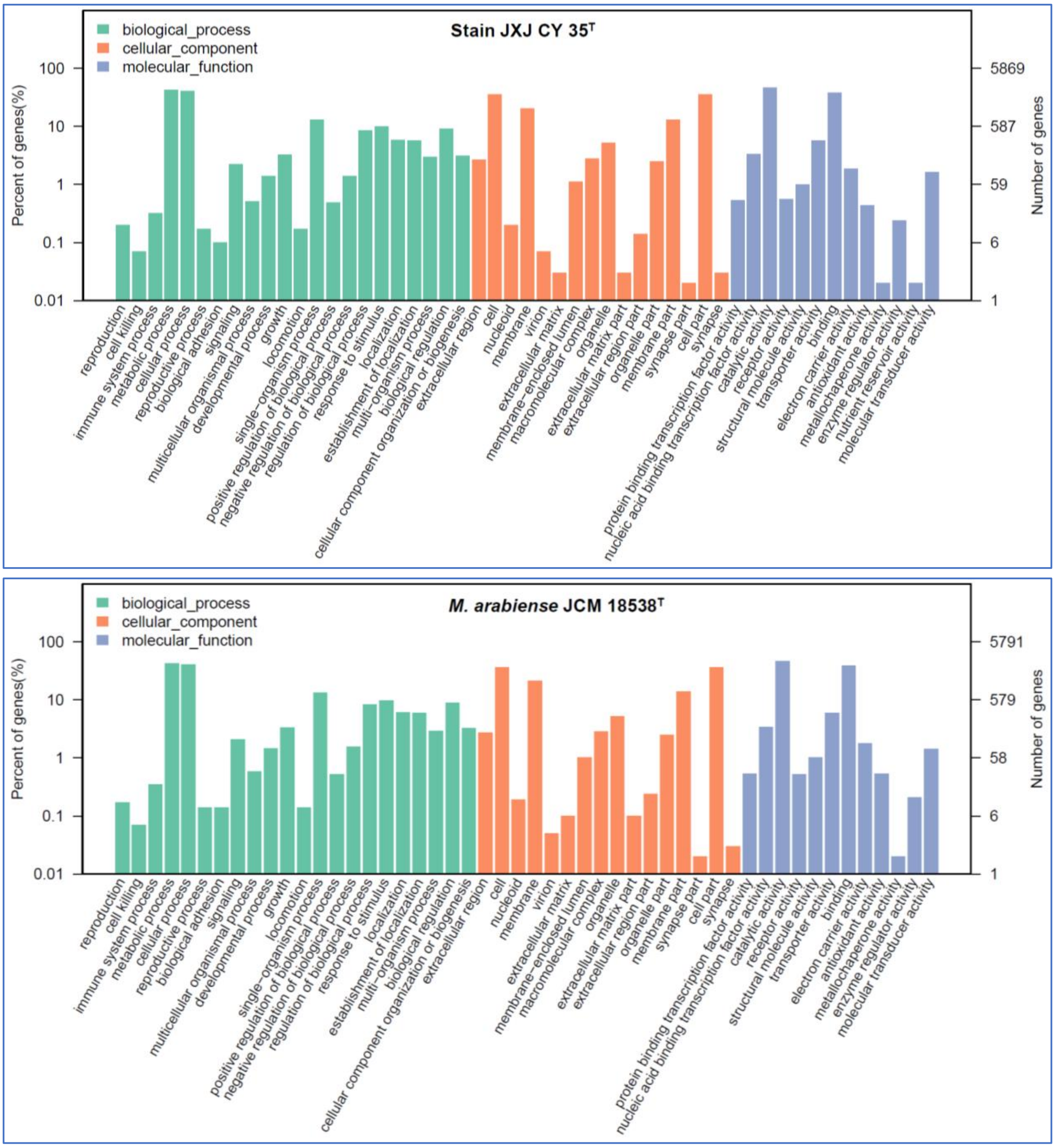

FIGURE S7 | Analysis of the GO functional classifications based on the genomes of stains JXJ CY 35^{T} and *M. arabiense* JCM 18538^{T}. (All data were from this study).

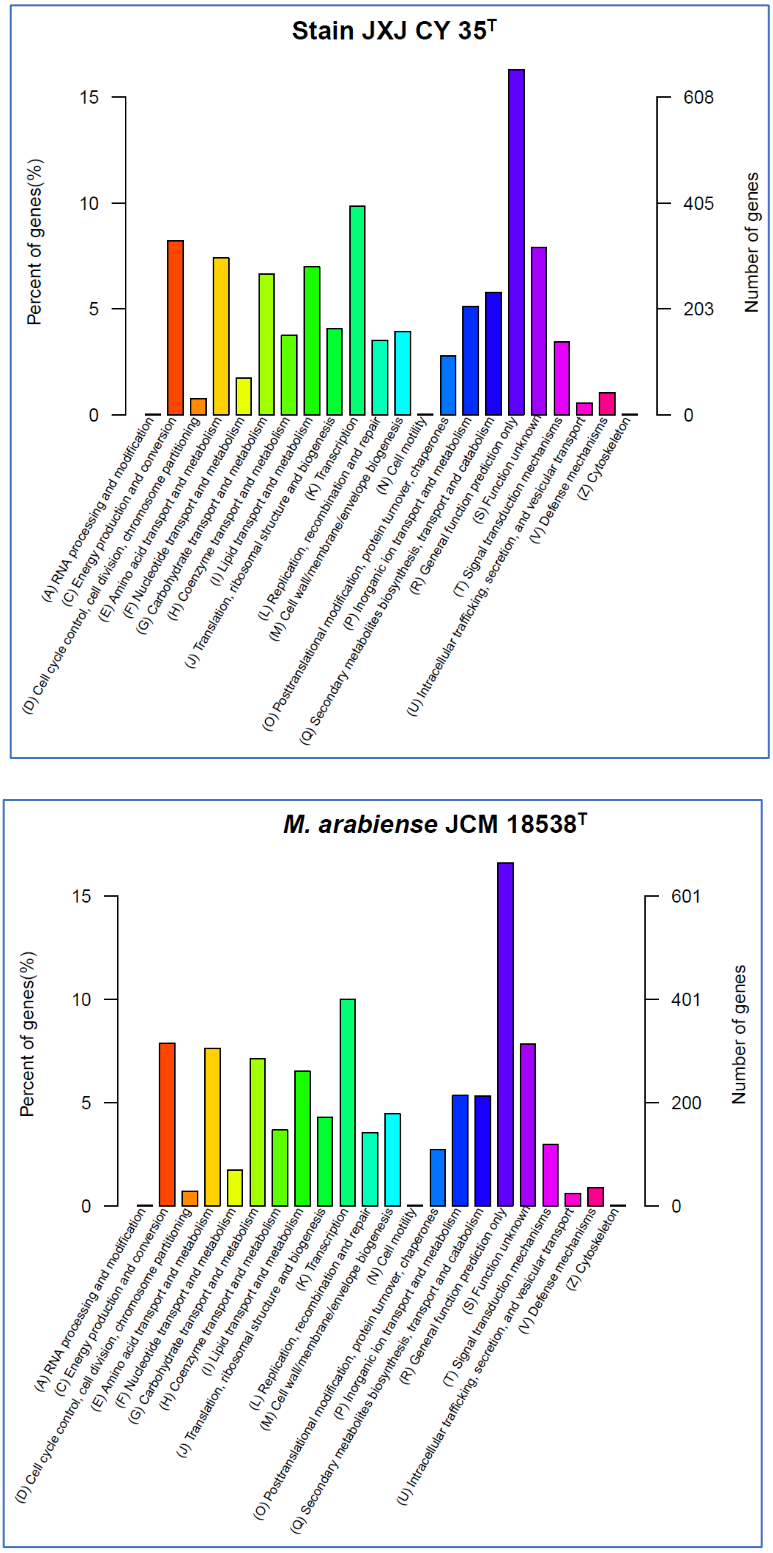

FIGURE S8 | Analysis of the COG functional classifications based on the genomes of stains JXJ CY 35^{T} and *M. arabiense* JCM 18538^{T}. (All data were from this study).

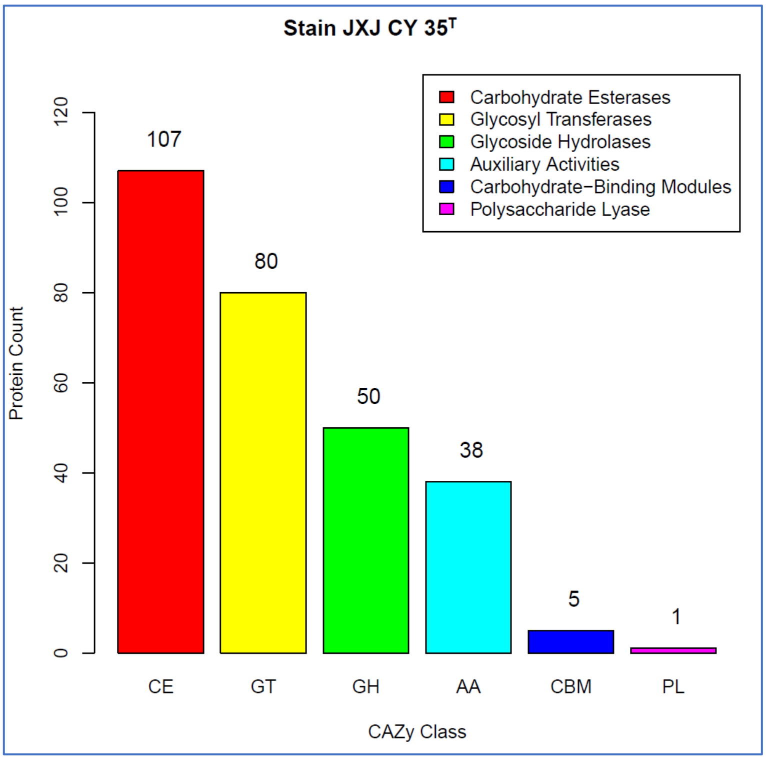

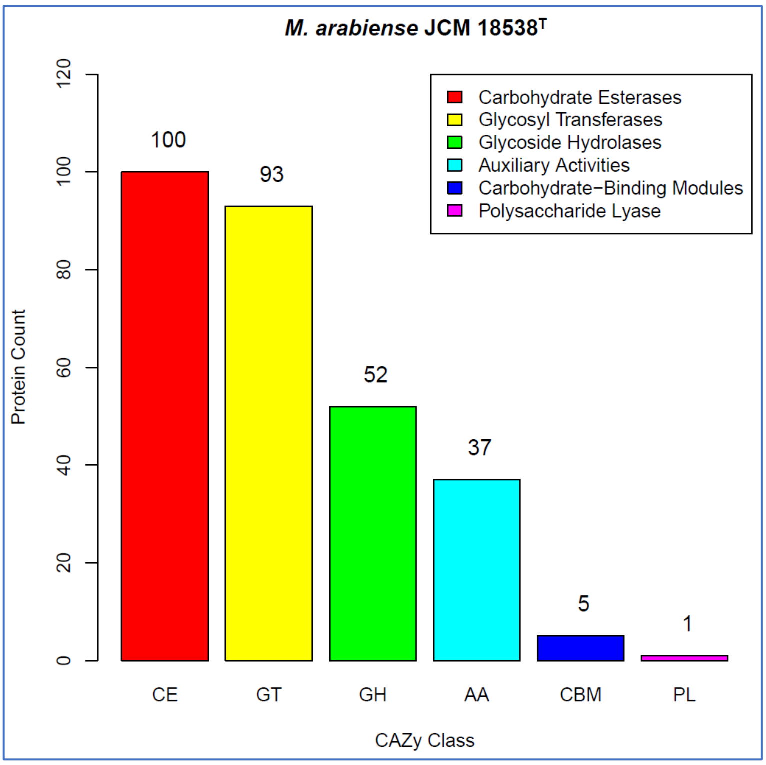

FIGURE S9 | Analysis of the CAZy functional classifications based on the genomes of stains JXJ CY 35^{T} and *M. arabiense* JCM 18538^{T}. (All data were from this study).

TABLE S1 | The gene annotation of strains JXJ CY 35^T and *M. arabiense* JCM 18538^T in various databases

Annotated database	Strain JXJ CY 35^T		*M. arabiense* JCM 18538^T	
	Number of Unigenes	Percentage (%)	Number of Unigenes	Percentage (%)
CDD	4718	80.39	4674	80.71
COG	4051	69.02	4009	69.23
NR	5774	98.38	5678	98.05
PFAM	4454	75.89	4396	75.91
Swissprot	4143	70.59	4085	70.54
TrEMBL	5767	98.26	5656	97.67
GO	4015	68.41	3984	68.80
KEGG	2460	41.92	2404	41.51
At least one database	5779	98.47	5686	98.19
All database	2214	37.72	2162	37.33
Total Unigenes	5869	100	5791	100

TABLE S2 | The possibly partial important genes or gene clusters for strain JXJ CY 35^{T} to adapt to the ecology of Maf

Level	GO	Term	Ontology	Gene_num	Gene_list
2	GO:0002376	immune system process	biological_process	19	PROKKA_04032, PROKKA_03356, PROKKA_03983, PROKKA_03979, PROKKA_03975, PROKKA_02774, PROKKA_03973, PROKKA_05172, PROKKA_00283, PROKKA_01612, PROKKA_03965, PROKKA_03980, PROKKA_04496, PROKKA_05271, PROKKA_04215, PROKKA_04798, PROKKA_00705, PROKKA_03981, PROKKA_04774
3	GO:0002682	regulation of immune system process	biological_process	18	PROKKA_04032, PROKKA_03356, PROKKA_03983, PROKKA_03979, PROKKA_03975, PROKKA_02774, PROKKA_03973, PROKKA_05172, PROKKA_00283, PROKKA_01612, PROKKA_03965, PROKKA_03980, PROKKA_04496, PROKKA_05271, PROKKA_04798, PROKKA_00705, PROKKA_03981, PROKKA_04774
3	GO:0002683	negative regulation of immune system process	biological_process	8	PROKKA_03965, PROKKA_03980, PROKKA_05271, PROKKA_03983, PROKKA_03979, PROKKA_03975, PROKKA_00705, PROKKA_03973
3	GO:0002684	positive regulation of immune system process	biological_process	6	PROKKA_04032, PROKKA_04496, PROKKA_03356, PROKKA_02774, PROKKA_01612, PROKKA_04774
3	GO:0006955	immune response	biological_process	19	PROKKA_04032, PROKKA_03356, PROKKA_03983, PROKKA_03979, PROKKA_03975, PROKKA_02774, PROKKA_03973, PROKKA_05172, PROKKA_00283, PROKKA_01612, PROKKA_03965, PROKKA_03980, PROKKA_04496, PROKKA_05271, PROKKA_04215, PROKKA_04798, PROKKA_00705, PROKKA_03981, PROKKA_04774
3	GO:0044110	growth involved in symbiotic interaction	biological_process	44	PROKKA_03532, PROKKA_05270, PROKKA_01249, PROKKA_04535, PROKKA_02386, PROKKA_05571, PROKKA_05578, PROKKA_05172, PROKKA_03973, PROKKA_01635, PROKKA_01479, PROKKA_01543, PROKKA_01250, PROKKA_02298, PROKKA_03980, PROKKA_03154, PROKKA_05570, PROKKA_01166, PROKKA_00657, PROKKA_02409, PROKKA_00571, PROKKA_03982, PROKKA_02720, PROKKA_04147, PROKKA_04677, PROKKA_00297, PROKKA_03979, PROKKA_02722,

					PROKKA_02213, PROKKA_03913, PROKKA_03028, PROKKA_04679, PROKKA_03218, PROKKA_02387, PROKKA_05572, PROKKA_02719, PROKKA_04991, PROKKA_02418, PROKKA_04798, PROKKA_05569, PROKKA_03534, PROKKA_02627, PROKKA_02723, PROKKA_02805
3	GO:0044111	development involved in symbiotic interaction	biological_process	4	PROKKA_05739, PROKKA_00057, PROKKA_00096, PROKKA_00047
3	GO:0052192	movement in environment of other organism involved in symbiotic interaction	biological_process	4	PROKKA_02061, PROKKA_02964, PROKKA_02252, PROKKA_00806
4	GO:0045087	innate immune response	biological_process	3	PROKKA_03965, PROKKA_05271, PROKKA_04215
4	GO:0050776	regulation of immune response	biological_process	18	PROKKA_04032, PROKKA_03356, PROKKA_03983, PROKKA_03979, PROKKA_03975, PROKKA_02774, PROKKA_03973, PROKKA_05172, PROKKA_00283, PROKKA_01612, PROKKA_03965, PROKKA_03980, PROKKA_04496, PROKKA_05271, PROKKA_04798, PROKKA_00705, PROKKA_03981, PROKKA_04774
4	GO:0050777	negative regulation of immune response	biological_process	8	PROKKA_03965, PROKKA_03980, PROKKA_05271, PROKKA_03983, PROKKA_03979, PROKKA_03975, PROKKA_00705, PROKKA_03973
4	GO:0050778	positive regulation of immune response	biological_process	6	PROKKA_04032, PROKKA_04496, PROKKA_03356, PROKKA_02774, PROKKA_01612, PROKKA_04774
4	GO:0051817	modification of morphology or physiology of other organism involved in symbiotic interaction	biological_process	19	PROKKA_04032, PROKKA_03356, PROKKA_03983, PROKKA_03979, PROKKA_03975, PROKKA_02774, PROKKA_05113, PROKKA_05172, PROKKA_00283, PROKKA_03973, PROKKA_01612, PROKKA_03965, PROKKA_03980, PROKKA_04496, PROKKA_00004, PROKKA_04798, PROKKA_00705, PROKKA_03981, PROKKA_04774

4	GO:0051828	entry into other organism involved in symbiotic interaction	biological_process	4	PROKKA_02061, PROKKA_02964, PROKKA_02252, PROKKA_00806
4	GO:0051883	killing of cells in other organism involved in symbiotic interaction	biological_process	1	PROKKA_00004
4	GO:0052047	interaction with other organism via secreted substance involved in symbiotic interaction	biological_process	1	PROKKA_00705
4	GO:0052173	response to defenses of other organism involved in symbiotic interaction	biological_process	71	PROKKA_04487, PROKKA_02005, PROKKA_03983, PROKKA_00175, PROKKA_05470, PROKKA_02503, PROKKA_03973, PROKKA_04613, PROKKA_00090, PROKKA_02858, PROKKA_03965, PROKKA_02006, PROKKA_03980, PROKKA_05592, PROKKA_04795, PROKKA_02638, PROKKA_04905, PROKKA_03154, PROKKA_03981, PROKKA_01390, PROKKA_00170, PROKKA_03236, PROKKA_03979, PROKKA_02774, PROKKA_05108, PROKKA_03028, PROKKA_00283, PROKKA_04496, PROKKA_00146, PROKKA_03929, PROKKA_00349, PROKKA_00092, PROKKA_03209, PROKKA_00674, PROKKA_04488, PROKKA_03534, PROKKA_02723, PROKKA_04774, PROKKA_03208, PROKKA_05301, PROKKA_03659, PROKKA_01249, PROKKA_01111, PROKKA_00569, PROKKA_01501, PROKKA_03366, PROKKA_05172, PROKKA_05028, PROKKA_00022, PROKKA_03709, PROKKA_00705, PROKKA_03658, PROKKA_01839, PROKKA_00938, PROKKA_04431, PROKKA_04032, PROKKA_05791, PROKKA_03356, PROKKA_04666, PROKKA_04677, PROKKA_01205, PROKKA_03975, PROKKA_01612, PROKKA_01862, PROKKA_04671, PROKKA_05010, PROKKA_03218, PROKKA_02622, PROKKA_02257, PROKKA_04798, PROKKA_05593

5	GO:0045088	regulation of innate immune response	biological_process	2	PROKKA_03965, PROKKA_05271
5	GO:0045824	negative regulation of innate immune response	biological_process	2	PROKKA_03965, PROKKA_05271
5	GO:0051801	cytolysis in other organism involved in symbiotic interaction	biological_process	1	PROKKA_00004
5	GO:0051806	entry into cell of other organism involved in symbiotic interaction	biological_process	4	PROKKA_02061, PROKKA_02964, PROKKA_02252, PROKKA_00806
5	GO:0051807	evasion or tolerance of defense response of other organism involved in symbiotic interaction	biological_process	17	PROKKA_03208, PROKKA_03236, PROKKA_04487, PROKKA_03979, PROKKA_03366, PROKKA_03973, PROKKA_00283, PROKKA_04613, PROKKA_05028, PROKKA_03980, PROKKA_02622, PROKKA_03709, PROKKA_00349, PROKKA_03209, PROKKA_04488, PROKKA_00170, PROKKA_01839
5	GO:0051816	acquisition of nutrients from other organism during symbiotic interaction	biological_process	2	PROKKA_05592, PROKKA_05593
5	GO:0051818	disruption of cells of other organism involved in symbiotic interaction	biological_process	1	PROKKA_00004
5	GO:0051832	avoidance of defenses of other organism involved in symbiotic interaction	biological_process	18	PROKKA_03208, PROKKA_03236, PROKKA_04487, PROKKA_03979, PROKKA_03366, PROKKA_03973, PROKKA_00283, PROKKA_04613, PROKKA_05028, PROKKA_03965, PROKKA_03980, PROKKA_02622, PROKKA_03709, PROKKA_00349, PROKKA_03209, PROKKA_04488, PROKKA_00170, PROKKA_01839
5	GO:0052048	interaction with host via secreted substance	biological_process	1	PROKKA_00705

		involved in symbiotic interaction			
5	GO:0052212	modification of morphology or physiology of other organism via secreted substance involved in symbiotic interaction	biological_process	1	PROKKA_00705
5	GO:0052248	modulation of programmed cell death in other organism involved in symbiotic interaction	biological_process	1	PROKKA_05113
5	GO:0052250	modulation of signal transduction in other organism involved in symbiotic interaction	biological_process	2	PROKKA_03973, PROKKA_03975
5	GO:0052255	modulation by organism of defense response of other organism involved in symbiotic interaction	biological_process	17	PROKKA_04032, PROKKA_03356, PROKKA_03983, PROKKA_03979, PROKKA_03975, PROKKA_02774, PROKKA_03973, PROKKA_05172, PROKKA_00283, PROKKA_01612, PROKKA_03965, PROKKA_03980, PROKKA_04496, PROKKA_00705, PROKKA_04798, PROKKA_03981, PROKKA_04774
5	GO:0052261	negative regulation by organism of defense response of other organism involved in symbiotic interaction	biological_process	1	PROKKA_03965
5	GO:0052552	modulation by organism of immune response of	biological_process	17	PROKKA_04032, PROKKA_03356,PROKKA_03983, PROKKA_03979, PROKKA_03975, PROKKA_02774, PROKKA_03973, PROKKA_05172, PROKKA_00283, PROKKA_01612, PROKKA_03965, PROKKA_03980,

		other organism involved in symbiotic interaction			PROKKA_04496, PROKKA_00705, PROKKA_04798, PROKKA_03981, PROKKA_04774
5	GO:0052555	positive regulation by organism of immune response of other organism involved in symbiotic interaction	biological_process	6	PROKKA_04032, PROKKA_04496, PROKKA_03356, PROKKA_02774, PROKKA_01612, PROKKA_04774
5	GO:0052561	negative regulation by organism of immune response of other organism involved in symbiotic interaction	biological_process	7	PROKKA_03965, PROKKA_03980, PROKKA_03983, PROKKA_03979, PROKKA_03975, PROKKA_00705, PROKKA_03973
5	GO:0052564	response to immune response of other organism involved in symbiotic interaction	biological_process	71	PROKKA_04487, PROKKA_02005, PROKKA_03983, PROKKA_00175, PROKKA_05470, PROKKA_02503, PROKKA_03973, PROKKA_04613, PROKKA_00090, PROKKA_02858, PROKKA_03965, PROKKA_02006, PROKKA_03980, PROKKA_05592, PROKKA_04795, PROKKA_02638, PROKKA_04905, PROKKA_03154, PROKKA_03981, PROKKA_01390, PROKKA_00170, PROKKA_03236, PROKKA_03979, PROKKA_02774, PROKKA_05108, PROKKA_03028, PROKKA_00283, PROKKA_04496, PROKKA_00146, PROKKA_03929, PROKKA_00349, PROKKA_00092, PROKKA_03209, PROKKA_00674, PROKKA_04488, PROKKA_03534, PROKKA_02723, PROKKA_04774, PROKKA_03208, PROKKA_05301, PROKKA_03659, PROKKA_01249, PROKKA_01111, PROKKA_00569, PROKKA_01501, PROKKA_03366, PROKKA_05172, PROKKA_05028, PROKKA_00022, PROKKA_03709, PROKKA_00705, PROKKA_03658, PROKKA_01839, PROKKA_00938, PROKKA_04431, PROKKA_04032, PROKKA_05791, PROKKA_03356, PROKKA_04666, PROKKA_04677, PROKKA_01205, PROKKA_03975, PROKKA_01612, PROKKA_01862,

					PROKKA_04671, PROKKA_05010, PROKKA_03218, PROKKA_02622, PROKKA_02257, PROKKA_04798, PROKKA_05593
6	GO:0051805	evasion or tolerance of immune response of other organism involved in symbiotic interaction	biological_process	17	PROKKA_03208, PROKKA_03236, PROKKA_04487, PROKKA_03979, PROKKA_03366, PROKKA_03973, PROKKA_00283, PROKKA_04613, PROKKA_05028, PROKKA_03980, PROKKA_02622, PROKKA_03709, PROKKA_00349, PROKKA_03209, PROKKA_04488, PROKKA_00170, PROKKA_01839
6	GO:0051833	suppression of defenses of other organism involved in symbiotic interaction	biological_process	1	PROKKA_03965
6	GO:0051834	evasion or tolerance of defenses of other organism involved in symbiotic interaction	biological_process	17	PROKKA_03208, PROKKA_03236, PROKKA_04487, PROKKA_03979, PROKKA_03366, PROKKA_03973, PROKKA_00283, PROKKA_04613, PROKKA_05028, PROKKA_03980, PROKKA_02622, PROKKA_03709, PROKKA_00349, PROKKA_03209, PROKKA_04488, PROKKA_00170, PROKKA_01839
6	GO:0052278	negative regulation by organism of cell-mediated immune response of other organism involved in symbiotic interaction	biological_process	5	PROKKA_03980, PROKKA_03979, PROKKA_03975, PROKKA_00705, PROKKA_03973
6	GO:0052294	modulation by organism of cell-mediated immune response of other organism involved in symbiotic interaction	biological_process	5	PROKKA_03980, PROKKA_03979, PROKKA_03975, PROKKA_00705, PROKKA_03973
6	GO:0052306	modulation by organism of innate immune	biological_process	4	PROKKA_03965, PROKKA_04798, PROKKA_00283, PROKKA_05172

		response in other organism involved in symbiotic interaction			
6	GO:0052309	negative regulation by organism of innate immune response in other organism involved in symbiotic interaction	biological_process	1	PROKKA_03965
6	GO:0052331	hemolysis in other organism involved in symbiotic interaction	biological_process	1	PROKKA_00004
6	GO:0052433	modulation by organism of apoptotic process in other organism involved in symbiotic interaction	biological_process	1	PROKKA_05113
6	GO:0052490	negative regulation by organism of programmed cell death in other organism involved in symbiotic interaction	biological_process	1	PROKKA_05113
6	GO:0052550	response to defense-related reactive oxygen species production by other organism involved in symbiotic interaction	biological_process	7	PROKKA_03236, PROKKA_04487, PROKKA_03709, PROKKA_03366, PROKKA_04613, PROKKA_04488, PROKKA_00170
6	GO:0052551	response to defense-related nitric oxide production by other	biological_process	3	PROKKA_00349, PROKKA_02622, PROKKA_03709

		organism involved in symbiotic interaction			
6	GO:0052553	modulation by symbiont of host immune response	biological_process	17	PROKKA_04032, PROKKA_03356, PROKKA_03983, PROKKA_03979, PROKKA_03975, PROKKA_02774, PROKKA_03973, PROKKA_05172, PROKKA_00283, PROKKA_01612, PROKKA_03965, PROKKA_03980, PROKKA_04496, PROKKA_00705, PROKKA_04798, PROKKA_03981, PROKKA_04774
6	GO:0052556	positive regulation by symbiont of host immune response	biological_process	6	PROKKA_04032, PROKKA_04496, PROKKA_03356, PROKKA_02774, PROKKA_01612, PROKKA_04774
6	GO:0052562	negative regulation by symbiont of host immune response	biological_process	7	PROKKA_03965, PROKKA_03980, PROKKA_03983, PROKKA_03979, PROKKA_03975, PROKKA_00705, PROKKA_03973
6	GO:0052572	response to host immune response	biological_process	71	PROKKA_04487, PROKKA_02005, PROKKA_03983, PROKKA_00175, PROKKA_05470, PROKKA_02503, PROKKA_03973, PROKKA_04613, PROKKA_00090, PROKKA_02858, PROKKA_03965, PROKKA_02006, PROKKA_03980, PROKKA_05592, PROKKA_04795, PROKKA_02638, PROKKA_04905, PROKKA_03154, PROKKA_03981, PROKKA_01390, PROKKA_00170, PROKKA_03236, PROKKA_03979, PROKKA_02774, PROKKA_05108, PROKKA_03028, PROKKA_00283, PROKKA_04496, PROKKA_00146, PROKKA_03929, PROKKA_00349, PROKKA_00092, PROKKA_03209, PROKKA_00674, PROKKA_04488, PROKKA_03534, PROKKA_02723, PROKKA_04774, PROKKA_03208, PROKKA_05301, PROKKA_03659, PROKKA_01249, PROKKA_01111, PROKKA_00569, PROKKA_01501, PROKKA_03366, PROKKA_05172, PROKKA_05028, PROKKA_00022, PROKKA_03709, PROKKA_00705, PROKKA_03658, PROKKA_01839, PROKKA_00938, PROKKA_04431, PROKKA_04032, PROKKA_05791, PROKKA_03356, PROKKA_04666, PROKKA_04677, PROKKA_01205, PROKKA_03975, PROKKA_01612, PROKKA_01862,

					PROKKA_04671, PROKKA_05010, PROKKA_03218, PROKKA_02622, PROKKA_02257, PROKKA_04798, PROKKA_05593
7	GO:0020012	evasion or tolerance of host immune response	biological_process	17	PROKKA_03208, PROKKA_03236, PROKKA_04487, PROKKA_03979, PROKKA_03366, PROKKA_03973, PROKKA_00283, PROKKA_04613, PROKKA_05028, PROKKA_03980, PROKKA_02622, PROKKA_03709, PROKKA_00349, PROKKA_03209, PROKKA_04488, PROKKA_00170, PROKKA_01839
7	GO:0051810	active evasion of immune response of other organism involved in symbiotic interaction	biological_process	7	PROKKA_03208, PROKKA_03980, PROKKA_03979, PROKKA_00283, PROKKA_03973, PROKKA_03209, PROKKA_05028
7	GO:0052083	negative regulation by symbiont of host cell-mediated immune response	biological_process	5	PROKKA_03980, PROKKA_03979, PROKKA_03975, PROKKA_00705, PROKKA_03973
7	GO:0052155	modulation by symbiont of host cell-mediated immune response	biological_process	5	PROKKA_03980, PROKKA_03979, PROKKA_03975, PROKKA_00705, PROKKA_03973
7	GO:0052167	modulation by symbiont of host innate immune response	biological_process	4	PROKKA_03965, PROKKA_04798, PROKKA_00283, PROKKA_05172
7	GO:0052170	negative regulation by symbiont of host innate immune response	biological_process	1	PROKKA_03965
7	GO:0052296	modulation by organism of microbe-associated molecular pattern-induced innate immune response in other	biological_process	2	PROKKA_04798, PROKKA_05172

		organism involved in symbiotic interaction			
7	GO:0052376	evasion or tolerance by organism of nitric oxide produced by other organism involved in symbiotic interaction	biological_process	3	PROKKA_00349, PROKKA_02622, PROKKA_03709
7	GO:0052385	evasion or tolerance by organism of reactive oxygen species produced by other organism involved in symbiotic interaction	biological_process	7	PROKKA_03236, PROKKA_04487, PROKKA_03709, PROKKA_03366, PROKKA_04613, PROKKA_04488, PROKKA_00170
8	GO:0042783	active evasion of host immune response	biological_process	7	PROKKA_03208, PROKKA_03980, PROKKA_03979, PROKKA_00283, PROKKA_03973, PROKKA_03209, PROKKA_05028
8	GO:0052157	modulation by symbiont of microbe-associated molecular pattern-induced host innate immune response	biological_process	2	PROKKA_04798, PROKKA_05172

TABLE S3 | The possibly partial important genes or gene clusters for strain JXJ CY 35^{T} to provide Maf with available P and N

Level	GO	Term	Ontology	Gene_num	Gene_list
6	GO:0016791	phosphatase activity	molecular_function	34	PROKKA_04980, PROKKA_01698, PROKKA_05726, PROKKA_03944, PROKKA_02834, PROKKA_03948, PROKKA_05654, PROKKA_03481, PROKKA_02348, PROKKA_00705, PROKKA_05788, PROKKA_00573, PROKKA_05944, PROKKA_01457, PROKKA_02275, PROKKA_02381, PROKKA_04723, PROKKA_03352, PROKKA_03605, PROKKA_04234, PROKKA_02881, PROKKA_05272, PROKKA_03056, PROKKA_05212, PROKKA_00577, PROKKA_05019, PROKKA_00304, PROKKA_03525, PROKKA_01008, PROKKA_01588, PROKKA_03140, PROKKA_04031, PROKKA_00566, PROKKA_03299
7	GO:0004035	alkaline phosphatase activity	molecular_function	2	PROKKA_03481, PROKKA_04234
4	GO:0009375	ferredoxin hydrogenase complex	cellular_component	2	PROKKA_01887, PROKKA_05841
5	GO:0009877	nodulation	biological_process	4	PROKKA_02878, PROKKA_01569, PROKKA_04267, PROKKA_03347
5	GO:0009399	nitrogen fixation	biological_process	8	PROKKA_00680, PROKKA_03221, PROKKA_01569, PROKKA_05728, PROKKA_01324, PROKKA_02741, PROKKA_02151, PROKKA_00686
3	GO:0006808	regulation of nitrogen utilization	biological_process	1	PROKKA_01479

TABLE S4 | The possibly partial important genes and gene clusters related to the nutrient exchange between strain JXJ CY 35^{T} and Maf

Level	GO	Term	Ontology	Gene_num	Gene_list
7	GO:0004574	oligo-1,6-glucosidase activity	molecular_function	2	PROKKA_01868, PROKKA_01061
7	GO:0008422	beta-glucosidase activity	molecular_function	3	PROKKA_01117, PROKKA_04298, PROKKA_05192
8	GO:0042973	glucan endo-1,3-beta-D-glucosidase activity	molecular_function	1	PROKKA_04298
6	GO:0004425	indole-3-glycerol-phosphate synthase activity	molecular_function	1	PROKKA_04731
6	GO:0042435	indole-containing compound biosynthetic process	biological_process	5	PROKKA_04731, PROKKA_04722, PROKKA_00649, PROKKA_04729, PROKKA_04036
6	GO:0043805	indolepyruvate ferredoxin oxidoreductase activity	molecular_function	2	PROKKA_04125, PROKKA_04237

6	GO:0080030	methyl indole-3-acetate esterase activity	molecular_function	12	PROKKA_01271, PROKKA_04586, PROKKA_05707, PROKKA_04293, PROKKA_01993, PROKKA_03276, PROKKA_03154, PROKKA_03183, PROKKA_04585, PROKKA_03028, PROKKA_01495, PROKKA_05393
4	GO:0009850	auxin metabolic process	biological_process	2	PROKKA_05608, PROKKA_04444
5	GO:0009851	auxin biosynthetic process	biological_process	2	PROKKA_05608, PROKKA_04444
6	GO:0009687	abscisic acid metabolic process	biological_process	2	PROKKA_00987, PROKKA_00110
7	GO:0009688	abscisic acid biosynthetic process	biological_process	2	PROKKA_00987, PROKKA_00110
5	GO:0009692	ethylene metabolic process	biological_process	1	PROKKA_01855
6	GO:0009693	ethylene biosynthetic process	biological_process	1	PROKKA_01855
4	GO:0015846	polyamine transport	biological_process	3	PROKKA_04273, PROKKA_00475, PROKKA_01931
5	GO:0015203	polyamine transmembrane transporter activity	molecular_function	10	PROKKA_05889, PROKKA_03094, PROKKA_01098, PROKKA_04271, PROKKA_04860, PROKKA_01323, PROKKA_04270, PROKKA_00477, PROKKA_02768, PROKKA_00474

6	GO:0015417	polyamine-transporting ATPase activity	molecular_function	4	PROKKA_04270, PROKKA_00477, PROKKA_04271, PROKKA_00474
7	GO:0006595	polyamine metabolic process	biological_process	15	PROKKA_03094, PROKKA_00285, PROKKA_04860, PROKKA_01323, PROKKA_02855, PROKKA_01555, PROKKA_05889, PROKKA_01320, PROKKA_03104, PROKKA_01098, PROKKA_04274, PROKKA_05017, PROKKA_02768, PROKKA_04863, PROKKA_01096
8	GO:0006596	polyamine biosynthetic process	biological_process	4	PROKKA_02855, PROKKA_01320, PROKKA_01555, PROKKA_00285
3	GO:0009349	riboflavin synthase complex	cellular_component	1	PROKKA_04600
4	GO:0004076	biotin synthase activity	molecular_function	1	PROKKA_04707
5	GO:0004746	riboflavin synthase activity	molecular_function	1	PROKKA_04598
5	GO:0009110	vitamin biosynthetic process	biological_process	57	PROKKA_05921, PROKKA_04599, PROKKA_04594, PROKKA_00526, PROKKA_02339, PROKKA_02034, PROKKA_02333, PROKKA_04702, PROKKA_00666, PROKKA_00690, PROKKA_02135, PROKKA_00357, PROKKA_04175, PROKKA_03601, PROKKA_00099, PROKKA_04598, PROKKA_02335, PROKKA_03398, PROKKA_04526, PROKKA_00519, PROKKA_04704, PROKKA_05758, PROKKA_00518, PROKKA_05002, PROKKA_00665, PROKKA_00527, PROKKA_02372, PROKKA_05170, PROKKA_01357, PROKKA_02701, PROKKA_00926, PROKKA_04600, PROKKA_00451, PROKKA_00514, PROKKA_02898, PROKKA_02340, PROKKA_00664, PROKKA_04010, PROKKA_01106, PROKKA_02036, PROKKA_01292, PROKKA_02015, PROKKA_01508, PROKKA_03737, PROKKA_04940, PROKKA_02035, PROKKA_02796, PROKKA_05705, PROKKA_03719, PROKKA_02101,

					PROKKA_00525, PROKKA_03718, PROKKA_04707, PROKKA_05769, PROKKA_02334, PROKKA_02709, PROKKA_00707
6	GO:0009102	biotin biosynthetic process	biological_process	8	PROKKA_05170, PROKKA_04940, PROKKA_02101, PROKKA_04707, PROKKA_04704, PROKKA_05002, PROKKA_04010, PROKKA_04702
6	GO:0009229	thiamine diphosphate biosynthetic process	biological_process	7	PROKKA_01508, PROKKA_04175, PROKKA_03737, PROKKA_03735, PROKKA_03719, PROKKA_03718, PROKKA_03733
6	GO:0010189	vitamin E biosynthetic process	biological_process	1	PROKKA_00357
6	GO:0042362	fat-soluble vitamin biosynthetic process	biological_process	1	PROKKA_00357
6	GO:0042364	water-soluble vitamin biosynthetic process	biological_process	56	PROKKA_05921, PROKKA_04599, PROKKA_04594, PROKKA_00526, PROKKA_02339, PROKKA_02034, PROKKA_02333, PROKKA_04702, PROKKA_00666, PROKKA_00690, PROKKA_02135, PROKKA_04175, PROKKA_03601, PROKKA_00099, PROKKA_04598, PROKKA_02335, PROKKA_03398, PROKKA_04526, PROKKA_00519, PROKKA_04704, PROKKA_05758, PROKKA_00518, PROKKA_05002, PROKKA_00665, PROKKA_00527, PROKKA_02372, PROKKA_05170, PROKKA_01357, PROKKA_02701, PROKKA_00926, PROKKA_04600, PROKKA_00451, PROKKA_00514, PROKKA_02898, PROKKA_02340, PROKKA_00664, PROKKA_04010, PROKKA_01106, PROKKA_02036, PROKKA_01292, PROKKA_02015, P ROKKA_01508, PROKKA_03737, PROKKA_04940, PROKKA_02035, PROKKA_02796, PROKKA_05705, PROKKA_03719, PROKKA_02101, PROKKA_00525, PROKKA_03718, PROKKA_04707, PROKKA_05769, PROKKA_02334, PROKKA_02709, PROKKA_00707

6	GO:0042724	thiamine-containing compound biosynthetic process	biological_process	6	PROKKA_01508, PROKKA_04175, PROKKA_03737, PROKKA_03719, PROKKA_03718, PROKKA_05769
6	GO:0042823	pyridoxal phosphate biosynthetic process	biological_process	12	PROKKA_04308, PROKKA_05490, PROKKA_00338, PROKKA_04474, PROKKA_00529, PROKKA_01011, PROKKA_04472, PROKKA_00513, PROKKA_04231, PROKKA_02709, PROKKA_01316, PROKKA_05348
7	GO:0009228	thiamine biosynthetic process	biological_process	6	PROKKA_01508, PROKKA_04175, PROKKA_03737, PROKKA_03719, PROKKA_03718, PROKKA_05769
7	GO:0009231	riboflavin biosynthetic process	biological_process	11	PROKKA_00099, PROKKA_05921, PROKKA_04598, PROKKA_04600, PROKKA_00451, PROKKA_04599, PROKKA_04594, PROKKA_03398, PROKKA_05758, PROKKA_01106, PROKKA_01292
7	GO:0009234	menaquinone biosynthetic process	biological_process	19	PROKKA_01706, PROKKA_03540, PROKKA_03516, PROKKA_01747, PROKKA_01742, PROKKA_01534, PROKKA_00826, PROKKA_01189, PROKKA_03528, PROKKA_03511, PROKKA_03506, PROKKA_03579, PROKKA_03499, PROKKA_00343, PROKKA_05018, PROKKA_05942, PROKKA_03319, PROKKA_01409, PROKKA_04881
7	GO:0042372	phylloquinone biosynthetic process	biological_process	1	PROKKA_01742
7	GO:0042819	vitamin B6 biosynthetic process	biological_process	2	PROKKA_02709, PROKKA_02701

TABLE S5 | The possibly partial important genes or gene clusters of strain JXJ CY 35^{T} related to signaling, signaling pathway and regulations

Level	GO	Term	Ontology	Gene_num	Gene_list
2	GO:0023052	signaling	biological_process	132	PROKKA_00446, PROKKA_03221, PROKKA_05790, PROKKA_00773, PROKKA_00065, PROKKA_03973, PROKKA_02354, PROKKA_02834, PROKKA_01543, PROKKA_01178, PROKKA_00260, PROKKA_02562, PROKKA_05645, PROKKA_00104, PROKKA_05120, PROKKA_00547, PROKKA_01399, PROKKA_05015, PROKKA_00772, PROKKA_03561, PROKKA_03994, PROKKA_05056, PROKKA_05664, PROKKA_02437, PROKKA_00823, PROKKA_04988, PROKKA_05665, PROKKA_05677, PROKKA_00322, PROKKA_00745, PROKKA_00046, PROKKA_01417, PROKKA_03563, PROKKA_00599, PROKKA_03624, PROKKA_00548, PROKKA_05604, PROKKA_01384, PROKKA_05662, PROKKA_04497, PROKKA_03574, PROKKA_01025, PROKKA_03001, PROKKA_03231, PROKKA_02769, PROKKA_05646, PROKKA_03446, PROKKA_05404, PROKKA_03501, PROKKA_00600, PROKKA_01153, PROKKA_00765, PROKKA_03790, PROKKA_01546, PROKKA_02128, PROKKA_05498, PROKKA_02722, PROKKA_00354, PROKKA_02624, PROKKA_03220, PROKKA_00400, PROKKA_00872, PROKKA_03822, PROKKA_04403, PROKKA_04823, PROKKA_02558, PROKKA_02937, PROKKA_00469, PROKKA_01016, PROKKA_01149, PROKKA_02630, PROKKA_02395, PROKKA_04444, PROKKA_00249, PROKKA_01024, PROKKA_05240, PROKKA_00392, PROKKA_03852, PROKKA_05612, PROKKA_03890, PROKKA_04908, PROKKA_00323, PROKKA_04404, PROKKA_04464, PROKKA_01547, PROKKA_04482, PROKKA_02833, PROKKA_03127, PROKKA_00871, PROKKA_05499, PROKKA_00321, PROKKA_03611, PROKKA_04245, PROKKA_02731, PROKKA_01657, PROKKA_02561, PROKKA_03623, PROKKA_05073, PROKKA_02723, PROKKA_01249, PROKKA_05222, PROKKA_02246, PROKKA_03875, PROKKA_03494, PROKKA_03467, PROKKA_04494, PROKKA_05405, PROKKA_04989, PROKKA_01250, PROKKA_05403, PROKKA_02291, PROKKA_04757, PROKKA_00098, PROKKA_02196, PROKKA_04814, PROKKA_01215, PROKKA_00504, PROKKA_03770, PROKKA_03975, PROKKA_01499, PROKKA_03447, PROKKA_05454, PROKKA_02304, PROKKA_00782, PROKKA_04751,

					PROKKA_03575, PROKKA_03002, PROKKA_05661, PROKKA_04433, PROKKA_03463, PROKKA_02759, PROKKA_02936
3	GO:0004871	signal transducer activity	molecular_function	95	PROKKA_00446, PROKKA_01698, PROKKA_03221, PROKKA_05790, PROKKA_02630, PROKKA_00773, PROKKA_00065, PROKKA_00249, PROKKA_02834, PROKKA_01543, PROKKA_01024, PROKKA_02562, PROKKA_03852, PROKKA_05645, PROKKA_00104, PROKKA_05120, PROKKA_00547, PROKKA_01399, PROKKA_00772, PROKKA_04404, PROKKA_00323, PROKKA_03561, PROKKA_01547, PROKKA_03994, PROKKA_05056, PROKKA_05664, PROKKA_02437, PROKKA_02833, PROKKA_04988, PROKKA_01339, PROKKA_05665, PROKKA_05499, PROKKA_00321, PROKKA_05677, PROKKA_02869, PROKKA_00322, PROKKA_05013, PROKKA_02731, PROKKA_02561, PROKKA_04768, PROKKA_01657, PROKKA_03623, PROKKA_00046, PROKKA_02723, PROKKA_01417, PROKKA_03563, PROKKA_00599, PROKKA_03624, PROKKA_04980, PROKKA_00548, PROKKA_01249, PROKKA_01384, PROKKA_05662, PROKKA_03574, PROKKA_03494, PROKKA_01025, PROKKA_03467, PROKKA_05405, PROKKA_04494, PROKKA_01250, PROKKA_04989, PROKKA_03001, PROKKA_05403, PROKKA_03231, PROKKA_05646, PROKKA_03446, PROKKA_02291, PROKKA_04757, PROKKA_05404, PROKKA_03501, PROKKA_04870, PROKKA_00600, PROKKA_02196, PROKKA_00765, PROKKA_01153, PROKKA_01546, PROKKA_05498, PROKKA_02722, PROKKA_00504, PROKKA_00354, PROKKA_02624, PROKKA_03447, PROKKA_01499, PROKKA_04751, PROKKA_02304, PROKKA_03220, PROKKA_03575, PROKKA_03002, PROKKA_03822, PROKKA_04403, PROKKA_05661, PROKKA_02937, PROKKA_02936, PROKKA_03463, PROKKA_00469
3	GO:0023051	regulation of signaling	biological_process	6	PROKKA_03790,PROKKA_03975,PROKKA_03890,PROKKA_03973,PROKKA_02304,PROKKA_01016
3	GO:0038023	signaling receptor activity	molecular_function	33	PROKKA_03624, PROKKA_00599, PROKKA_03221, PROKKA_00548, PROKKA_00773, PROKKA_00065, PROKKA_02834, PROKKA_01543, PROKKA_03467, PROKKA_01024, PROKKA_04989, PROKKA_01250, PROKKA_02562, PROKKA_05646, PROKKA_03446, PROKKA_05404, PROKKA_00323, PROKKA_01547, PROKKA_03994, PROKKA_02722, PROKKA_00354, PROKKA_02304, PROKKA_01339, PROKKA_03575, PROKKA_05665,

					PROKKA_03002, PROKKA_05499, PROKKA_05677, PROKKA_05013, PROKKA_04403, PROKKA_05661, PROKKA_02937, PROKKA_03463
3	GO:0043190	ATP-binding cassette (ABC) transporter complex	cellular_component	7	PROKKA_02657, PROKKA_02862, PROKKA_02514, PROKKA_03222, PROKKA_02551, PROKKA_02685, PROKKA_01996
5	GO:0009306	protein secretion	biological_process	8	PROKKA_00643, PROKKA_01102, PROKKA_01104, PROKKA_02213, PROKKA_02770, PROKKA_01480, PROKKA_00558, PROKKA_02703
3	GO:0044700	single organism signaling	biological_process	132	PROKKA_00446, PROKKA_03221, PROKKA_05790, PROKKA_00773, PROKKA_00065, PROKKA_03973, PROKKA_02354, PROKKA_02834, PROKKA_01543, PROKKA_01178, PROKKA_00260, PROKKA_02562, PROKKA_05645, PROKKA_00104, PROKKA_05120, PROKKA_00547, PROKKA_01399, PROKKA_05015, PROKKA_00772, PROKKA_03561, PROKKA_03994, PROKKA_05056, PROKKA_05664, PROKKA_02437, PROKKA_00823, PROKKA_04988, PROKKA_05665, PROKKA_05677, PROKKA_00322, PROKKA_00745, PROKKA_00046, PROKKA_01417, PROKKA_03563, PROKKA_00599, PROKKA_03624, PROKKA_00548, PROKKA_05604, PROKKA_01384, PROKKA_05662, PROKKA_04497, PROKKA_03574, PROKKA_01025, PROKKA_03001, PROKKA_03231, PROKKA_02769, PROKKA_05646, PROKKA_03446, PROKKA_05404, PROKKA_03501, PROKKA_00600, PROKKA_01153, PROKKA_00765, PROKKA_03790, PROKKA_01546, PROKKA_02128, PROKKA_05498, PROKKA_02722, PROKKA_00354, PROKKA_02624, PROKKA_03220, PROKKA_00400, PROKKA_00872, PROKKA_03822, PROKKA_04403, PROKKA_04823, PROKKA_02558, PROKKA_02937, PROKKA_00469, PROKKA_01016, PROKKA_01149, PROKKA_02630, PROKKA_02395, PROKKA_04444, PROKKA_00249, PROKKA_01024, PROKKA_05240, PROKKA_00392, PROKKA_03852, PROKKA_05612, PROKKA_03890, PROKKA_04908, PROKKA_00323, PROKKA_04404, PROKKA_04464, PROKKA_01547, PROKKA_04482, PROKKA_02833, PROKKA_03127, PROKKA_00871, PROKKA_05499, PROKKA_00321, PROKKA_03611, PROKKA_04245, PROKKA_02731, PROKKA_01657,

					PROKKA_02561, PROKKA_03623, PROKKA_05073, PROKKA_02723, PROKKA_01249, PROKKA_05222, PROKKA_02246, PROKKA_03875, PROKKA_03494, PROKKA_03467, PROKKA_04494, PROKKA_05405, PROKKA_04989, PROKKA_01250, PROKKA_05403, PROKKA_02291, PROKKA_04757, PROKKA_00098, PROKKA_02196, PROKKA_04814, PROKKA_01215, PROKKA_00504, PROKKA_03770, PROKKA_03975, PROKKA_01499, PROKKA_03447, PROKKA_05454, PROKKA_02304, PROKKA_00782, PROKKA_04751, PROKKA_03575, PROKKA_03002, PROKKA_05661, PROKKA_04433, PROKKA_03463, PROKKA_02759, PROKKA_02936
4	GO:0003001	generation of a signal involved in cell-cell signaling	biological_process	1	PROKKA_03790
4	GO:0004888	transmembrane signaling receptor activity	molecular_function	2	PROKKA_05404, PROKKA_02304
4	GO:0007165	signal transduction	biological_process	130	PROKKA_00446, PROKKA_03221, PROKKA_05790, PROKKA_00773, PROKKA_00065, PROKKA_03973, PROKKA_02354, PROKKA_02834, PROKKA_01543, PROKKA_01178, PROKKA_00260, PROKKA_02562, PROKKA_05645, PROKKA_00104, PROKKA_05120, PROKKA_00547, PROKKA_01399, PROKKA_05015, PROKKA_00772, PROKKA_03561, PROKKA_03994, PROKKA_05056, PROKKA_05664, PROKKA_02437, PROKKA_00823, PROKKA_04988, PROKKA_05665, PROKKA_05677, PROKKA_00322, PROKKA_00745, PROKKA_00046, PROKKA_01417, PROKKA_03563, PROKKA_00599, PROKKA_03624, PROKKA_00548, PROKKA_05604, PROKKA_01384, PROKKA_05662, PROKKA_04497, PROKKA_03574, PROKKA_01025, PROKKA_03001, PROKKA_03231, PROKKA_02769, PROKKA_05646, PROKKA_03446, PROKKA_05404, PROKKA_03501, PROKKA_00600, PROKKA_01153, PROKKA_00765, PROKKA_01546, PROKKA_02128, PROKKA_05498, PROKKA_02722, PROKKA_00354, PROKKA_02624, PROKKA_03220, PROKKA_00400,

					PROKKA_00872, PROKKA_03822, PROKKA_04403, PROKKA_04823, PROKKA_02558, PROKKA_02937, PROKKA_00469, PROKKA_01016, PROKKA_01149, PROKKA_02630, PROKKA_02395, PROKKA_00249, PROKKA_04444, PROKKA_01024, PROKKA_05240, PROKKA_00392, PROKKA_03852, PROKKA_05612, PROKKA_03890, PROKKA_04908, PROKKA_00323, PROKKA_04404, PROKKA_04464, PROKKA_01547, PROKKA_04482, PROKKA_02833, PROKKA_03127, PROKKA_00871, PROKKA_05499, PROKKA_00321, PROKKA_03611, PROKKA_04245, PROKKA_02731, PROKKA_01657, PROKKA_02561, PROKKA_03623, PROKKA_05073, PROKKA_02723, PROKKA_01249, PROKKA_05222, PROKKA_02246, PROKKA_03875, PROKKA_03494, PROKKA_03467, PROKKA_04494, PROKKA_05405, PROKKA_04989, PROKKA_01250, PROKKA_05403, PROKKA_02291, PROKKA_04757, PROKKA_00098, PROKKA_02196, PROKKA_04814, PROKKA_01215, PROKKA_00504, PROKKA_03770, PROKKA_03975, PROKKA_01499, PROKKA_03447, PROKKA_05454, PROKKA_00782, PROKKA_04751, PROKKA_03575, PROKKA_03002, PROKKA_05661, PROKKA_04433, PROKKA_03463, PROKKA_02759, PROKKA_02936
4	GO:0007267	cell-cell signaling	biological_process	2	PROKKA_03790, PROKKA_02304
4	GO:0009966	regulation of signal transduction	biological_process	4	PROKKA_03890, PROKKA_03973, PROKKA_01016, PROKKA_03975
4	GO:0035637	multicellular organismal signaling	biological_process	1	PROKKA_02304
4	GO:0044501	modulation of signal transduction in other organism	biological_process	2	PROKKA_03973, PROKKA_03975

4	GO:0048500	signal recognition particle	cellular_component	1	PROKKA_01477
5	GO:0000160	phosphorelay signal transduction system	biological_process	7	PROKKA_04245, PROKKA_00773, PROKKA_05404, PROKKA_04757, PROKKA_01543, PROKKA_01016, PROKKA_01024
5	GO:0005786	signal recognition particle, endoplasmic reticulum targeting	cellular_component	1	PROKKA_01477
5	GO:0007166	cell surface receptor signaling pathway	biological_process	8	PROKKA_00260, PROKKA_03611, PROKKA_04464, PROKKA_05604, PROKKA_05015, PROKKA_02246, PROKKA_03875, PROKKA_00823
5	GO:0009755	hormone-mediated signaling pathway	biological_process	2	PROKKA_04908, PROKKA_04444
5	GO:0023014	signal transduction by phosphorylation	biological_process	29	PROKKA_03624, PROKKA_00599, PROKKA_03221, PROKKA_00548, PROKKA_00773, PROKKA_00065, PROKKA_02834, PROKKA_01543, PROKKA_03467, PROKKA_01024, PROKKA_04989, PROKKA_01250, PROKKA_02562, PROKKA_05646, PROKKA_03446, PROKKA_05404, PROKKA_00323, PROKKA_01547, PROKKA_03994, PROKKA_02722, PROKKA_00354, PROKKA_03575, PROKKA_03002, PROKKA_05499, PROKKA_05665, PROKKA_04403, PROKKA_05661, PROKKA_02937, PROKKA_03463

5	GO:0023061	signal release	biological_process	1	PROKKA_03790
5	GO:0035556	intracellular signal transduction	biological_process	90	PROKKA_00446, PROKKA_01149, PROKKA_05790, PROKKA_02630, PROKKA_02395, PROKKA_02354, PROKKA_00249, PROKKA_01178, PROKKA_00260, PROKKA_05240, PROKKA_03852, PROKKA_00392, PROKKA_05645, PROKKA_00104, PROKKA_05612, PROKKA_05120, PROKKA_00547, PROKKA_01399, PROKKA_00772, PROKKA_04404, PROKKA_03561, PROKKA_04464, PROKKA_03994, PROKKA_05056, PROKKA_04482, PROKKA_05664, PROKKA_02437, PROKKA_02833, PROKKA_03127, PROKKA_04988, PROKKA_00871, PROKKA_00321, PROKKA_00322, PROKKA_02731, PROKKA_02561, PROKKA_01657, PROKKA_00745, PROKKA_03623, PROKKA_00046, PROKKA_05073, PROKKA_02723, PROKKA_01417, PROKKA_03563, PROKKA_05222, PROKKA_01249, PROKKA_01384, PROKKA_05662, PROKKA_04497, PROKKA_02246, PROKKA_03574, PROKKA_03494, PROKKA_01025, PROKKA_03467, PROKKA_05405, PROKKA_04494, PROKKA_03001, PROKKA_05403, PROKKA_03231, PROKKA_02291, PROKKA_04757, PROKKA_03501, PROKKA_00098, PROKKA_00600, PROKKA_04814, PROKKA_00765, PROKKA_01153, PROKKA_02196, PROKKA_01215, PROKKA_01546, PROKKA_02128, PROKKA_05498, PROKKA_00504, PROKKA_00354, PROKKA_03770, PROKKA_02624, PROKKA_01499, PROKKA_03447, PROKKA_05454, PROKKA_04751, PROKKA_00782, PROKKA_03220, PROKKA_00400, PROKKA_00872, PROKKA_03822, PROKKA_02558, PROKKA_04433, PROKKA_00469, PROKKA_02759, PROKKA_02936, PROKKA_03463
5	GO:0052027	modulation by symbiont of host signal transduction pathway	biological_process	2	PROKKA_03973, PROKKA_03975
5	GO:0052250	modulation of signal transduction	biological_process	2	PROKKA_03973, PROKKA_03975

		in other organism involved in symbiotic interaction			
5	GO:0070297	regulation of phosphorelay signal transduction system	biological_process	1	PROKKA_01016
5	GO:0097190	apoptotic signaling pathway	biological_process	4	PROKKA_05240, PROKKA_04823, PROKKA_02769, PROKKA_00400
6	GO:0006465	signal peptide processing	biological_process	1	PROKKA_03240
6	GO:0007167	enzyme linked receptor protein signaling pathway	biological_process	3	PROKKA_02246, PROKKA_00260, PROKKA_04464
6	GO:0007186	G-protein coupled receptor signaling pathway	biological_process	4	PROKKA_05015, PROKKA_03875, PROKKA_03611, PROKKA_00823

6	GO:0007264	small GTPase mediated signal transduction	biological_process	2	PROKKA_02128, PROKKA_03127
6	GO:0008543	fibroblast growth factor receptor signaling pathway	biological_process	3	PROKKA_02246, PROKKA_00260, PROKKA_04464
6	GO:0008631	intrinsic apoptotic signaling pathway in response to oxidative stress	biological_process	2	PROKKA_05240, PROKKA_00400
6	GO:0016055	Wnt receptor signaling pathway	biological_process	1	PROKKA_05604
6	GO:0038179	neurotrophin signaling pathway	biological_process	3	PROKKA_02246, PROKKA_00260, PROKKA_04464
6	GO:0043401	steroid hormone mediated signaling pathway	biological_process	2	PROKKA_04908, PROKKA_04444

6	GO:0048017	inositol lipid-mediated signaling	biological_process	3	PROKKA_02246, PROKKA_00260, PROKKA_04464
6	GO:0097193	intrinsic apoptotic signaling pathway	biological_process	2	PROKKA_05240, PROKKA_00400
7	GO:0007169	transmembrane receptor protein tyrosine kinase signaling pathway	biological_process	3	PROKKA_02246, PROKKA_00260, PROKKA_04464
7	GO:0007205	protein kinase C-activating G-protein coupled receptor signaling pathway	biological_process	4	PROKKA_05015, PROKKA_03875, PROKKA_03611, PROKKA_00823
7	GO:0007265	Ras protein signal transduction	biological_process	1	PROKKA_02128
7	GO:0009742	brassinosteroid mediated signaling pathway	biological_process	2	PROKKA_04908, PROKKA_04444

7	GO:0043491	protein kinase B signaling cascade	biological_process	5	PROKKA_01215, PROKKA_00260, PROKKA_04464, PROKKA_03770, PROKKA_02246
7	GO:0048011	neurotrophin TRK receptor signaling pathway	biological_process	3	PROKKA_02246, PROKKA_00260, PROKKA_04464
7	GO:0048015	phosphatidylinositol-mediated signaling	biological_process	3	PROKKA_02246, PROKKA_00260, PROKKA_04464
8	GO:0008286	insulin receptor signaling pathway	biological_process	3	PROKKA_02246, PROKKA_00260, PROKKA_04464
8	GO:0038127	ERBB signaling pathway	biological_process	3	PROKKA_02246, PROKKA_00260, PROKKA_04464
9	GO:0007173	epidermal growth factor receptor signaling pathway	biological_process	3	PROKKA_02246, PROKKA_00260, PROKKA_04464
